# Attributing medical spending to conditions: A comparison of methods

**DOI:** 10.1371/journal.pone.0237082

**Published:** 2020-08-10

**Authors:** Kaushik Ghosh, Irina Bondarenko, Kassandra L. Messer, Susan T. Stewart, Trivellore Raghunathan, Allison B. Rosen, David M. Cutler

**Affiliations:** 1 The National Bureau of Economic Research, Cambridge, Massachusetts, United States of America; 2 Institute for Social Research and Department of Biostatistics, University of Michigan, Ann Arbor, Michigan, United States of America; 3 Population and Quantitative Health Sciences, University of Massachusetts Medical School, Worcester, Massachusetts, United States of America; 4 Department of Economics, Harvard University, Cambridge, Massachusetts, United States of America; University of Utah, UNITED STATES

## Abstract

To understand the cost burden of medical care it is essential to partition medical spending into conditions. Two broad strategies have been used to measure disease-specific spending. The first attributes each medical claim to the condition that physicians list as its cause. The second decomposes total spending for a person over a year to their cumulative set of health conditions. Traditionally, this has been done through regression analysis. This paper has two contributions. First, we develop a new cost attribution method to attribute spending to conditions using a more flexible attribution approach, based on propensity score analysis. Second, we compare the propensity score approach to the claims-based approach and the regression approach in a common set of beneficiaries age 65 and older in the 2009 Medicare Current Beneficiary Survey. Our estimates show that the three methods have important differences in spending allocation and that the propensity score model likely offers the best theoretical and empirical combination.

## Introduction

For many purposes, it is important to attribute medical spending to particular conditions. For example, researchers and policy makers often ask questions such as: Does spending align with the burden of disease, or are the two independent [1]? How much of medical spending growth is associated with the rising prevalence of chronic disease [2–4]? Each of these questions requires a condition-based look at spending.

The fundamental difficulty with attributing spending to conditions is comorbidities. If all people had only one medical condition at a time, it would be easy to measure condition-based spending. When people have multiple conditions, however, this becomes more difficult. If a person has a heart attack from which recovery is slow, is the extra spending during the post-acute period a result of an abnormally slow recovery, or might it result instead from pre-existing mental illness, which makes following a recommended medication and lifestyle pattern more difficult? One needs some type of disease attribution methodology to answer this question.

There are two common methods in the literature to attribute spending to medical conditions: a claim-based method and a regression-based method. In the claims-based method, spending is assigned to conditions based on the diagnosis codes listed on the claim. Claims-based methods have been employed since the 1960s [5,6]. The claims-based method is problematic for claims that may result from multiple listed conditions. For example, an analyst faced with the example above would need to decide how to attribute spending to heart disease and mental illness–even assuming that both are listed on the claim. The regression model typically relates total spending over the calendar year to the full set of medical conditions the person has. However, these regressions usually have a large unexplained component–the constant term of the regression. It is not clear what condition to assign that spending to.

This paper has two goals. Our first goal is to develop a method of spending attribution that is more robust than the claims and regression methods. The second goal is to compare the three methods of allocating spending to conditions using data from the 2009 Medicare Current Beneficiary Survey. We propose a three-step propensity score methodology to do this. The first step uses a propensity score method to compare people with a condition to observably similar people without that condition. In the second step, the difference in spending between those individuals is used as a first-pass estimate of the condition-specific cost. In the third step, we model total spending as a function of attributed costs and a number of comorbidities and utilization of medical services. We do this to fit the pattern of high spenders more accurately.

### Conceptual framework

Our goal is to estimate how much it costs to treat different conditions. For example, if medical spending increased from one year to the next, how much of that was accounted for by heart disease, cancer, joint pain, or other conditions? Because spending is controlled by physicians, one way to do this is to ask physicians directly how much they spend on different conditions. However, asking physicians directly about this allocation is not helpful because each specialist treats a separate medical condition and so they do not know how much the condition they treat compares to other conditions in influencing spending. Further, physicians often have different ideas or frameworks on how to associate dollars and services depending on medical severity. For example, the cost of treating heart disease may depend on whether the patient also has cancer or not, and physicians may not be able to give answers in this fashion. Thus, another method is needed.

There are different ways in the literature to allocate dollars to medical conditions. The two common ones are the claims and regression approaches. The claims method attributes each medical claim to the condition that physicians list as its cause. Implicitly, it solves the attribution problem through the lens of the physician’s major diagnostic decision. The second decomposes total spending for a person over a year to their cumulative set of health conditions based on a regression relating spending to the full set of medical conditions. Each of these methods has their own shortcomings, which we explain below. Because of this, we propose a propensity score method to allocate spending to medical conditions by stratifying individuals based on comorbid conditions, socio-economic and demographic factors, and then comparing spending for people with and without the relevant condition.

Having three different estimates, we then consider how to judge their relative merit. We discuss several features of each model: how easily it can be implemented; whether all dollars are allocated to conditions; how well the model explains spending at the individual level; and how the model performs in out-of-sample predictions. For the latter criterion, the key test we perform is to add up condition-specific spending to the individual level based on the conditions that a person has. We compare the distribution of predicted individual-level spending to actual individual-level spending. A better model will fit the distribution of individual spending with less error.

## Data and methods

Our primary data source is the 2009 Cost and Use sample of the Medicare Current Beneficiary Survey (MCBS). We restrict our sample to the population aged 65 and older since the MCBS is nationally representative for the older population. The MCBS has information on survey-reported medical events, supplemented by Medicare claims. Our use of this data was determined by the Institutional Review Boards of NBER and Harvard University to be exempt from human subjects protections because it is secondary data not collected for this study, and is provided such that subjects cannot be identified.

We adjust the MCBS in several ways to make spending and disease prevalence nationally representative. Because the MCBS has incomplete or no medical claims information for beneficiaries enrolled in Health Maintenance Organizations (HMOs), we reweight the fee-for-service population to compensate for this exclusion [7]. The total number of observations in the traditional Medicare sample is 6,200 compared to about 10,000 when including those in Medicare Advantage. Second, we make an adjustment for the difference between MCBS survey spending and national spending estimates of personal health care spending, along the lines of previous literature [7,8].

The disease prevalence rates from MCBS claims for some conditions (for example, hypertension and hyperlipidemia) are lower than those observed from self-reports in surveys such as the National Health and Nutritional Examination Survey (NHANES), which also collects laboratory results. We use the NHANES to calibrate the prevalence of conditions in MCBS using a multiple imputation procedure. We term the resulting conditions “calibrated medical conditions” [9,10]. Section A4 in S1 Appendix gives an overview of the calibration method and for more details refer to Raghunathan, et al. [10]. The imputation procedure produces five imputed data sets. The medical conditions are imputed at the individual level. In our empirical analysis, we estimate the models for each of the five imputed data sets and combine the means and standard errors from the five imputed data sets using standard combination methods.

An important consideration is how many conditions to model. We developed a classification schema for medical conditions building upon the Agency for Healthcare Research and Quality’s (AHRQ) Clinical Classification Software (CCS), which aggregates the 14,000+ ICD-9-CM diagnosis codes and 3,900+ ICD-9-CM procedure codes into a smaller number of clinically meaningful, mutually exclusive categories [11]. To define medical conditions, we began with the 259 CCS categories delineated by the AHRQ. The physicians in our group determined that a few conditions identified in larger CCS categories should be stand-alone disease categories because of their clinical significance in the elderly (mostly mental health). For example, while the CCS has a single “mood disorders” category, we separated this into two separate groups–depression and bipolar disorder.

Not all of the AHRQ-delineated conditions have a high prevalence in the elderly (for example, the prevalence of pregnancy-related conditions is very low). After combining and disaggregating as noted above, we determined *ex-ante* a set of 105 relevant conditions. In a preliminary analysis, several of the calibrated conditions had too low a prevalence to meaningfully estimate their cost in the elderly. Thus, for the purpose of cost estimation, we combine these 105 conditions into 78 conditions with a larger sample size (see S1 Table in S1 Appendix): 71 diagnosed conditions; 3 undiagnosed conditions (high cholesterol, high blood pressure, and diabetes), and 4 cancer screening variables (colon cancer, cervical cancer, breast cancer and prostate cancer). We estimate costs for each of these 78 conditions. For presentation purposes, we combine the results into 17 multi-level CCS categories.

### Methods for attributing spending to conditions

There are two fundamental methodologies in the literature for attributing spending to conditions: a claims-based method that assigns spending for particular claims to one or more conditions coded as the reason for the medical visit; and a regression method that relates total spending over a period of time to a set of medical conditions and uses that to decompose spending into attributable conditions. We discuss briefly each method in turn, highlighting how we implement each. Technical details and additional results are provided in S1 Appendix. Before we present our methodologies, we note one feature of the analysis. We seek to estimate the partial effect of each condition on spending, controlling for all other conditions that a person has–e.g., the effect of diabetes controlling for cardiovascular disease. That differs from the total effect that spending on a disease might have, for example since diabetes increases the risk of cardiovascular disease.

#### Claims based approach

The claims-based approach attributes spending from each medical bill to the conditions listed on the bill. If one condition is listed, all of the dollars are attributed to that condition. If there is more than one condition, the spending is divided among the conditions. Numerous studies implement the claims-based method in slightly different ways [5,6,12–15]. Our analysis follows Thorpe et al [4] as much as possible, making additional adjustments as appropriate.

In each set of claims files (hospital inpatient, hospital outpatient, carrier/professional providers, hospice, home health, skilled nursing facility and durable medical equipment), we first identify claims with only one listed condition and average the costs for a condition using those claims. For claims with multiple listed conditions, we then allocate costs to each reported condition based on how expensive that condition was when it was the only listed condition relative to all the conditions listed. For example, if heart disease is twice as expensive as diabetes when each is the only condition on a claim, then spending on a claim with both heart disease and diabetes listed as conditions is allocated two-thirds to heart disease and one-third to diabetes.

Not all claims sources have claims on which large shares have only one condition. For example, most hospital records have more than one condition. In this case, we use the same methodology, but the weights we use to disaggregate spending are based on claims listing a condition as the primary diagnosis, not the sole diagnosis. Another limitation of the claims approach is that the prescription drug data in MCBS have no diagnosis codes listed. To attribute prescription drug spending to conditions, we identify all medical conditions the person was treated for in 2009 using inpatient, outpatient, skilled nursing facility, carrier, hospice, home health and durable medical equipment claims. Each condition is counted once and assigned a DRG weight based on inpatient admissions with that as the primary condition. We apportion total prescription drug spending for the year based on the share of the total DRG weights accounted for by each condition. These methods are described in more detail in S1 Appendix. Using DRG weights based on inpatient admissions to allocate drug claims may be problematic for pharmaceuticals taken for chronic disease. However, it is not obvious that there is a better way to do this.

Finally, for the 5.3% of beneficiaries who have dollar amounts in the personal summary file(s), but have no medical claims, we use the calibrated claims from the NHANES instead of actual claims and assign dollars to calibrated medical conditions using the same methodology as for pharmaceutical costs. The result of this process is a complete decomposition of medical spending into the 78 categories described above.

#### Regression based approach

The second method of attributing spending to medical conditions is to use regression analysis. The regression model relates total spending over the year to the full set of conditions that a person has. The coefficients are then used to find spending for each condition [16–21]. If the equation is linear in spending (e.g., the dependent variable is the dollar value of spending during the year), the coefficients on the condition indicators indicate the spending attributable to each condition. If the model is non-linear, as for example when researchers relate the logarithm of spending plus $1 to conditions, a transformation is needed to back out condition-specific costs in dollars.

To implement the regression models, we include as independent variables 78 health conditions (which are comorbid conditions) and other covariates that are expected to influence medical costs. These include: age, gender, education (8^th^ grade or less, 9^th^-12^th^ no diploma, high school diploma, associate/some college and college degree or higher), military service, race/ethnicity dummies (non-Hispanic white, non-Hispanic black, Hispanic and other), marital status (married, widowed, divorced/separated, never married), current smoking, ever smoking, pneumonia shot, flu shot, hysterectomy, poverty level (4 categories based on the Federal Poverty level (FPL), and any private health insurance coverage during the calendar year. These also include measures of health: difficulty lifting (10 lbs), difficulty stooping, difficulty walking (1/4 mi. or 2–3 blocks), difficulty dressing, difficulty eating, self-rated health (excellent, very good, good, fair, poor), self-reported health status compared to one year ago (better, same, worse). Other covariate variables include: any use of hearing aid, days in a long-term care facility (such as a nursing home, rehabilitation hospital, mental health facility, or institution for the developmentally disabled), count of inpatient hospital nights, count of inpatient hospital stays, height, weight, and probability of death in the given calendar year.

We estimate the spending equation for each of the 5 multiply imputed data sets and then average the estimates. To identify the best fitting regression model, we explored several variants of Generalized Linear Models (GLM) using both one and two-part models [22–31].

Some studies make an additional adjustment for people with no medical spending, for example using a two-part model: one equation for the probability of positive spending and the second for the amount spent [27–29]. We estimate two-part models assuming the probability that a beneficiary has positive health care spending is a probit. For people with non-zero spending, an OLS or GLM regression is run with the same set of covariates as in the probit model [29].

We estimated total spending with a Gamma distribution and a log link function. We also estimated log (spending+1) as the dependent variable with a Gaussian distribution and an identity link. Other models include a cubic root model (cubic root of spending), and Box-Cox model. The diagnostic plots are described in detail in S1 Appendix.

The log specification assumes that each disease has a multiplicative effect on spending. An additional transformation is needed to turn this into dollars, and to ensure that the average dollars spent will match the known total. We follow a methodology described by Trogdon et al. [18]; the equations are in S1 Appendix.

A common concern that arises in the regression model is that conditions can be associated with decreased spending–in a linear model, nothing constrains each condition to have a positive cost. Generally, negative coefficients are ascribed to diagnostic mismeasurement [32,33]. When relatively healthy people see physicians, physicians still need some condition to code as the cause of the visit. In such circumstances, the physician may code a relatively benign condition (hypertension or high cholesterol, for example) that is common in the population and straightforward to justify. For patients with a variety of conditions, in contrast, physicians may focus on more acute conditions and not record risk factors that have little immediate impact on health. Alternatively, negative coefficients might result from overfitting, or from high collinearity among included variables.

Some studies address the issue of negative coefficients by forcing the coefficients to be positive. In practice, however, this effectively amounts to making some conditions have zero spending. Other studies address the issue by defining hierarchical categories to pull out difficult cases. For example, rather than coding for hypertension, the regression might include categories for ‘hypertension without other conditions’, ‘hypertension with heart disease’, and so on [30]. Conditions are often defined so that spending on every condition is positive. For our purposes, we do not wish to change the set of conditions across estimation methodologies. For conditions that had negative spending coefficients for 1–4 of the five multiply imputed data sets, we treat those variables as missing and average the coefficients over the remaining replicates. As shown in S5 Fig in S1 Appendix, two of the condition groups have negative coefficients for all five of the replicates: deep vein thrombosis and acute renal failure. These are relatively rare conditions (6% and 9% respectively), but the claims-based approach attributed $2.2 billion and $7.7 billion to them respectively. We set spending to zero for those two conditions [33,34].

#### Propensity score method

The limitations of the claims-based method and the regression method noted above and in the results motivate us to develop a more flexible model. Our estimation methodology consists of three steps.

*Step 1*: *Form otherwise similar groups of people with and without each condition*.

We start by forming groups of people without each condition that are demographically and clinically similar to people with each condition. For example, if people with heart disease tend to have diabetes, we want to match people with heart disease and diabetes to those with diabetes but without heart disease, so that we can estimate the impact of heart disease on spending with the effect of diabetes controlled for. We do this using propensity score analysis. We fit separate logistic regression models for each condition on the other 77 medical conditions, with a few exceptions. The other covariates used in the propensity score models are the same demographic, socioeconomic and health status covariates used in the regression approach (see regression section for details). The only exception is that we exclude other medical conditions when they have a deterministic or extremely tight correlation with the condition of the interest; for example, people with breast cancer almost never have prostate cancer (S5 Table in S1 Appendix shows propensity score model exclusions).

We also estimate the probability of death in a given year and then use the estimated probability of death as a covariate in the propensity score models. Death cannot be included directly, as there was small fraction of decedents (roughly 5%) in any calendar year. We do not include spending as a covariate in the models. We perform the Hosmer-Lemeshow goodness of fit test to assess our logistic regression models used in computing the propensity scores. Furthermore, we compare the distribution of covariates for those with the condition and those without within each propensity score stratum in order to ensure a balance of covariates within each stratum. We find good overlap in the covariate profiles between cases and controls to allow us to properly estimate the attributable costs for each condition. S6 Table in S1 Appendix is the cohort balance table for Acute Myocardial Infarction. We fail to reject equality of the prevalence in almost all cases.

After computing the propensity scores for a disease category, subjects are grouped into strata of equal size based on their propensity scores. In general, we use five strata per condition, though conditions with a small number of cases are grouped into fewer strata to avoid situations where a small number of cases has a large impact on the results (see S6 Fig in S1 Appendix).

*Step 2*: *Estimate the average difference in spending for individuals with and without each disease*.

We estimate the mean difference in expenditures between cases and controls within each stratum and then average these differences across strata to obtain an estimate of healthcare expenditure attributable to that condition. This analysis is done for each of the five multiply imputed data sets and the results are averaged across data sets. S7 Table in S1 Appendix shows the first-stage cost estimates for all 78 medical conditions.

*Step 3*: *Adjust condition spending to match national totals and better match the variability in individual spending*.

The results derived in step 2 have two limitations. First, the weighted sum of spending across conditions is not guaranteed to add to national totals. This is a difference between the propensity score model and the regression model, where the latter necessarily has mean predicted spending equal to the mean actual spending. No such constraint is imposed by the propensity score model. Second, the propensity score model does not fit the distribution of spending at the individual level well. Similar to the regression approach, there are not enough high and low spenders in the predicted data compared to the actual data. This is not an issue of small samples. Rather, the gap is more fundamental. High spenders have many of the same conditions as average spenders, but these conditions spiral out of control for some and become very expensive.

To demonstrate this, S7 Fig in S1 Appendix plots the difference between predicted costs based on summing conditions for each person and actual costs based on various measures of utilization: the number of comorbidities; whether the person lives in an institution; the number of hospitalizations; and whether the person survived the year in each case; the predictions are farther off for people who are sicker. For example, when the number of comorbidities is low–roughly 3 or fewer–there is no systematic difference between predicted cost and observed cost. However, as the number of comorbidities increases, the first stage cost progressively underestimates the observed cost.

To address these issues, we relate observed cost at the individual level (denoted AC_i_) to predicted cost formed by adding up condition-specific costs based on the actual conditions of each person (denoted *SC_i_*). The regression is of the form:

$$E({AC}_{i})={SC}_{i}{(\alpha }_{0}+{K_{l}\alpha }_{l}).$$

The vector K consists of variables that pick up high spending: the number of comorbidities, that number squared, an indicator for any hospitalization, number of nights in the hospital, number of hospital admissions, number of days institutionalized, survival in the indicated year, number of months survived for those who are deceased, and number of outpatient claims. S3 Table in S1 Appendix shows the coefficient of the adjusted costs model. All enter as we expect: higher utilization helps close the gap between actual and predicted spending.

To integrate these adjustments into the estimated cost of specific conditions, we first aggregate the adjustment factors for each disease by taking the average of the individuals with that condition. We then apply this disease-specific adjustment factor to the estimates from step 2. The results are estimates of spending at the condition level that are guaranteed to sum to total spending and that track the individual distributions of spending as well as possible. S7 Table in S1 Appendix shows the adjusted cost and the disease-specific adjustment factor for each of the 78 medical conditions.

The adjustment that we make to better match actual and simulated spending could be made with the regression model as well, in which case the regression model would better fit the distribution of high spenders. For consistency with the existing literature, however, we present the regression model without this adjustment.

## Results: Comparison of cost attribution methods

Table 1 reports the total attributed spending by multi-level CCS categories. In the claims-based approach, the diseases of the circulatory system are the most expensive category, accounting for 23% of all spending, followed by the diseases of the nervous system and sense organs and diseases of the musculoskeletal system and connective tissue. Together, the top five disease categories account for nearly 60% of all personal health care spending. At the level of specific conditions, the most expensive conditions are acute events with expensive and often prolonged hospitalizations: acute myocardial infarction, cardiac arrest, hematologic cancers, lung cancer, and acute renal failure (see S4 Table in S1 Appendix).

**Table 1:** Estimated total cost in 2009 (billions of 2010 US $) using different methods.

	Total Attributed Cost (Billions)			% of Total Spending		
Multi-level CCS	Claims	Regression	Propensity Score	Claims	Regression	Propensity Score
7-Diseases of the circulatory system	$148.1	$119.3	$144.8	23.0%	18.5%	22.5%
16-Other conditions	41.3	121.1	83.4	6.4%	18.8%	13.0%
8-Diseases of the respiratory system	53.4	35.5	62.4	8.3%	5.5%	9.7%
3-Endocrine; nutritional; and metabolic disease	49.2	48.9	54.9	7.6%	7.6%	8.5%
6-Diseases of the nervous system	64.0	43.0	44.9	9.9%	6.7%	7.0%
10-Diseases of the genitourinary system	38.1	28.6	41.6	5.9%	4.4%	6.5%
5-Mental Illness	30.8	18.3	39.5	4.8%	2.8%	6.1%
15-Injury and poisoning	27.5	26.1	37.0	4.3%	4.1%	5.7%
13-Diseases of the musculoskeletal system	62.2	41.6	36.1	9.7%	6.5%	5.6%
4-Diseases of the blood	17.7	12.9	32.8	2.7%	2.0%	5.1%
9-Diseases of the digestive system	28.1	33.1	28.5	4.4%	5.1%	4.4%
2-Neoplasms	41.4	27.6	22.5	6.4%	4.3%	3.5%
1-Infectious and parasitic diseases	20.3	12.6	14.4	3.2%	2.0%	2.2%
12-Diseases of the skin and subcutaneous tissue	12.6	12.7	7.9	2.0%	2.0%	1.2%
14 -Congenital Anomalies & perinatal	4.6	2.4	4.6	0.7%	0.4%	0.7%
11-Complications of pregnancy; childbirth, etc.	1.4	0.5	-0.2	0.2%	0.1%	0.0%
17-Screening	3.0	1.9	-11.5	0.5%	0.3%	-1.8%
Other covariates (including intercept)	X	57.7	X	X	9.0%	X
**Total**	$643.7	$643.7	$643.7	100.0%	100.0%	100.0%

The table is sorted from highest to lowest attributed total cost in the propensity score method. The multi-level CCS category “16-Other Condition” includes Signs and Symptoms ($62.97 billion) and Residual, unclassified, all other E codes ($20.46 billion).X = Not Applicable.

The second column of Table 1 shows spending by multi-level CCS category from the regression approach; S4 Table in S1 Appendix shows the spending attributed to each of the 78 medical conditions. The regression estimates differ in many ways from the claims-based estimates. Other conditions (Signs and Symptoms; Residual, unclassified and all other Ecodes) are much more important in the regression approach, accounting for 19% of spending compared to 6% in the claims approach. Subsequent analysis suggests that this is largely due to high attributed spending for signs and symptoms, for example nausea or fainting without cause. Circulatory disease is second in spending (19%).

The third most expensive condition is not an actual condition; it is the unexplained component–the constant term and other covariates. This picks up spending that the regression does not attribute to any medical condition. Unfortunately for the regression approach, this is very large–$58 billion, or 9% of total spending. Note that the constant term includes the constant from the regression and also the combined impact of the omitted categories of all other covariates which are not medical conditions, such as age, education, and race. Because we believe that these factors should be controlled for in a spending model, we choose not to estimate a model without any covariates. Note also that the constant term is not a result of omitted conditions. All conditions are included in our regression model, either directly or through residual categories.

The third column of Table 1 shows spending by multi-level CCS category from the propensity score; S4 Table in S1 Appendix shows the spending attributed to each of the 78 medical conditions. Diseases of the circulatory system are the most expensive category, accounting for 23% of personal health spending. The next most expensive categories are “other conditions”; diseases of the respiratory system, endocrine, nutritional and metabolic diseases and immunity disorders; and diseases of the nervous system and sense organs. Together, these disease groups account for almost 61% of personal health spending.

Two of the conditions (cancer screening; complications of pregnancy, childbirth, and the puerperium) are estimated to have negative spending. The results for cancer screening are intriguing; they possibly suggest that cancer screening saves money. Another alternative, however, is that cancer screening is more likely to be conducted in people who are otherwise healthy, and this picks up unmeasured health status.

Our data do not allow us to fully differentiate between these two hypotheses, but some guesses are possible. The MCBS data contain a self-report of health status relative to others of the same age. Controlling for other factors, people receiving cancer screening have higher self-reported health than those that do not. On average, 52% of the elderly Medicare receiving cancer screening report excellent or very good health as compared to 42% among beneficiaries receiving no cancer screening. Also, only 3% of the beneficiaries receiving cancer screening report poor heath as compared to 7% receiving no screening. Thus, we suspect the negative spending may be related to better overall health.

**Fig 1:**
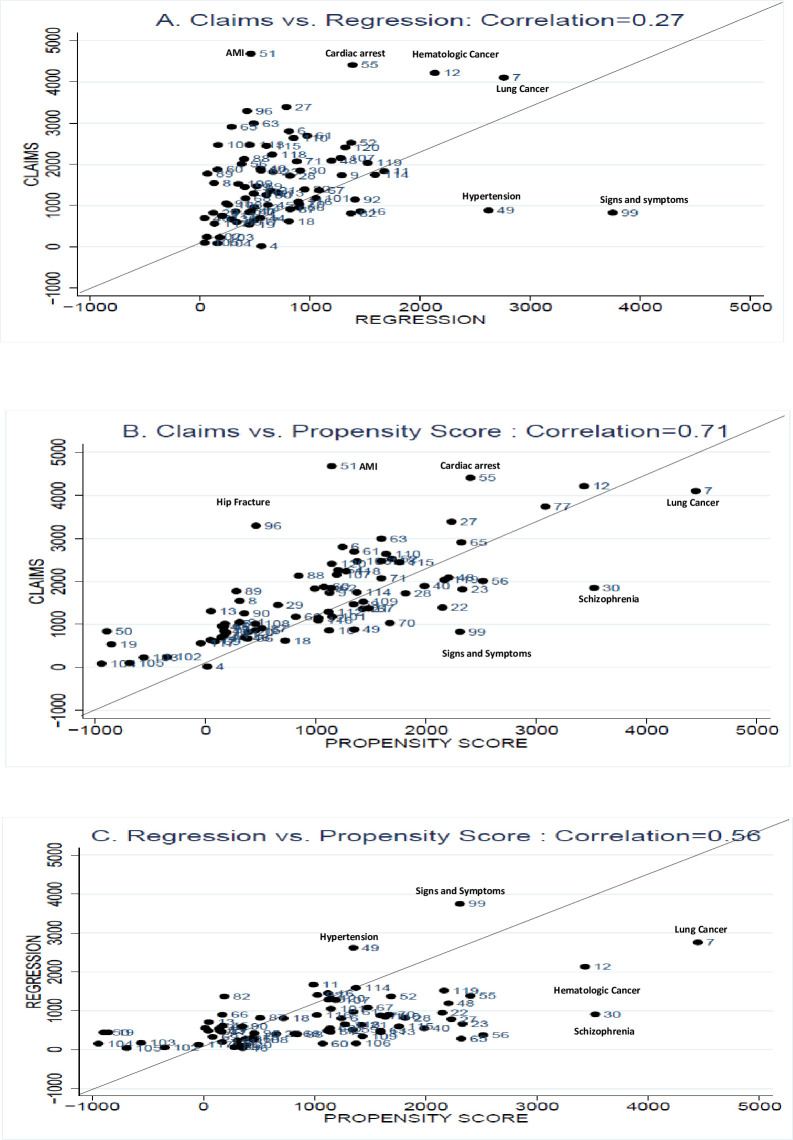
Average attributed cost using three attribution methods. Acute Renal Failure and Deep Vein Thrombosis (DVT) had negative coefficients in regression model and no cost attributed. As a result, they are not included in the correlation and also in Panel A and Panel C. Numbers labeling data points refer to conditions listed in Table A1 in S1 Appendix.

Fig 1 shows two-way scatter plots of average attributed cost for the claims-based, regression-based, and propensity score approaches. Panel A compares the claims-based approach and the regression approach. Panel B compares the claims-based approach and propensity score approaches, and panel C compares the regression and propensity score approaches. The correlation between average disease-specific spending using the different approaches is reasonably high: 0.27 between the claims-based approach and the regression approach, 0.71 between the claims-based approach and propensity score approach, and 0.56 between the regression approach and the propensity score approach. The figures show that some large and significant outliers influence the correlations.

The average attributed cost from the claims-based method is higher for most of the conditions than for the other two approaches. This is not surprising; the claims approach estimates the cost of the condition that resulted in the claim, whereas the propensity score and regression methods estimate cost attributed to the condition regardless of whether it is specified in the claim or remains latent–for example, the cost of a stroke that has a claim in a given year is likely to be higher than a past stroke whose residual effects are still present. A corollary of this is that the effective number of conditions for each claim is higher in the regression and propensity score approaches. In general, acute medical conditions like acute myocardial infarction, cardiac arrest, hematologic cancer, and hip fracture have significantly higher average costs in the claims-based method as compared to the regression and propensity score methods.

Not surprisingly, the average attributed cost from the regression method and the propensity score method have much greater accord than either one has with the claim-based approach. Most of the conditions are around the 45-degree line, with a few exceptions that are off-diagonal. The major differences are that conditions like lung cancer, hematologic cancer, and schizophrenia have much higher average attributed cost in the propensity score method as compared to the regression-based method. On the other hand, hypertension and signs and symptoms have significantly higher attributed costs in the regression-based approach.

Intuition suggests that expensive, rare diseases are likely to generate high spenders or outliers from the cost analysis perspective. The regression method, by assuming parametric models and distributions, may smooth over these extreme values, whereas the non-parametric propensity score approach gives higher weight to these outliers. On the other hand, attributed cost estimation for very prevalent and less severe conditions such as hypertension and signs and symptoms may be influenced by collinearity with other conditions, which could be more of an issue for the regression approach.

**Fig 2:**
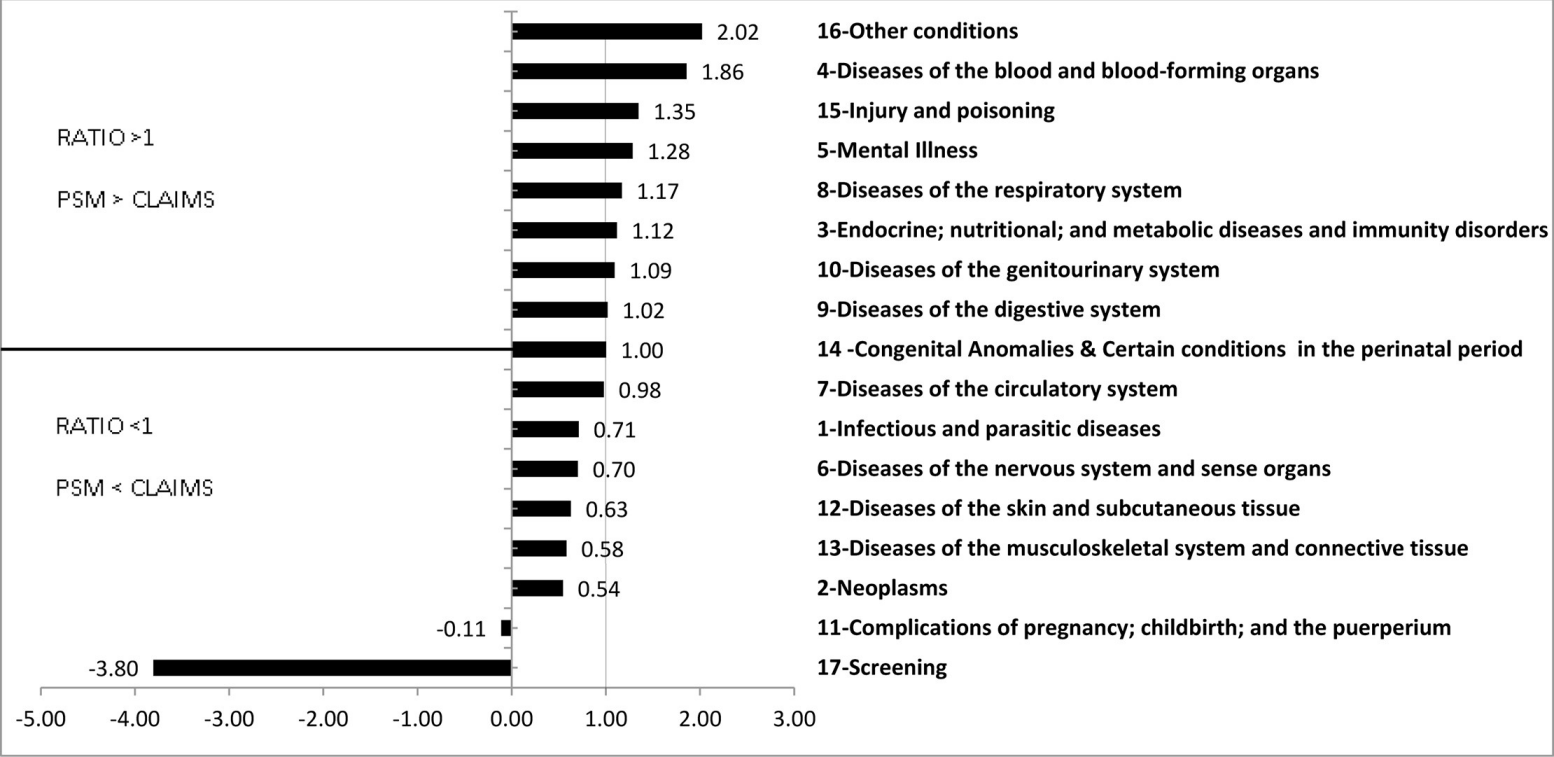
Ratio of total cost by major CCS categories: Propensity score vs. claims. Figure shows the ratio of total costs for the propensity score method to the claims-attribution method. The ratios are based on total attributed dollars reported in Table 1. Ratio >1 implies PSM method attributes more total spending than claims method, and ratio <1 implies vice-versa. Other Conditions (top bar) include Signs and Symptoms; Residual, unclassified and all other E codes. The propensity score method attributes negative spending to various cancer screenings and complications of pregnancy, making the ratios negative.

In addition to average spending per condition, we also care about total spending on each condition–taking account of prevalence as well as cost per condition. Fig 2 shows the ratio of total costs for the propensity score method to the claims-attribution method, using the total dollars displayed in Table 1. A ratio >1 implies that the propensity score method attributes more spending than claims, and a ratio <1 implies the opposite. The biggest ratios (>1) are for diseases of the blood and blood-forming organs (largely anemia), injury and poisoning, and mental illness. Some of these are important comorbid conditions. Anemia can generally be treated cheaply, but its presence indicates a more severe form of disease. Thus, it is natural that people with anemia spend a lot more than people without. Similarly, mental illness has been shown in many studies to be a significant risk factor for spending [17].

A flip occurs at diseases of circulatory system, where the claims approach attributes more dollars than the propensity score approach. Two ratios are negative–cancer screening; and complications of menopause, pregnancy, childbirth, and the puerperium. The propensity score method attributes negative spending to those conditions whereas the claims-based approach attributes positive spending. In the elderly population, complications of menopause, pregnancy, childbirth, and the puerperium are largely due to menopause. Women coded for this may be relatively healthy–if there were other, more severe conditions, they would likely be coded. Screening may be negative for the same reason (because healthier people get screened) or because screening prevents more expensive diseases.

Using all three metrics, disease specific spending in the elderly population is very concentrated. In the claims-based approach, the top 5 (10) conditions account for 20% (36%) of total dollars. The shares are 45% (61%) for the regression approach and 29% (46%) for the propensity score model.

In the propensity score approach, the most expensive conditions generally cost about $3,000 to $4,000 annually. For example, the cost of lung cancer ranges from $2,800 to $4,400. This is true using all methods and seems unusual given *ex-ante* expectations. For example, the cost of almost any chemotherapy regimen will exceed the few thousand dollars that we estimate cancer to cost. The issue here is that not all of the prevalent cases are incident cases. Imagine that a person was diagnosed with lung cancer in 2008, receives the bulk of their care in that year and has a few visits for monitoring in 2009. We will record that person as (correctly) having lung cancer in 2009, even though the case is not new. Further, lung cancer spending will be relatively low in 2009 unless the person has a cancer recurrence. The net effect will be relatively low average spending per case.

If one were developing a model of lung cancer cost effectiveness, the spending we estimate would be of limited use. For such a model, one would want to know spending by the phase of cancer: the acute phase, maintenance phase, and (possibly) terminal phase. In studying the decomposition of total spending into conditions, however, the estimate we have is very relevant: it correctly indicates the average amount spent per person treated with cancer in the given year for a representative cross-section of the population, as well as the total dollars in the population.

**Fig 3:**
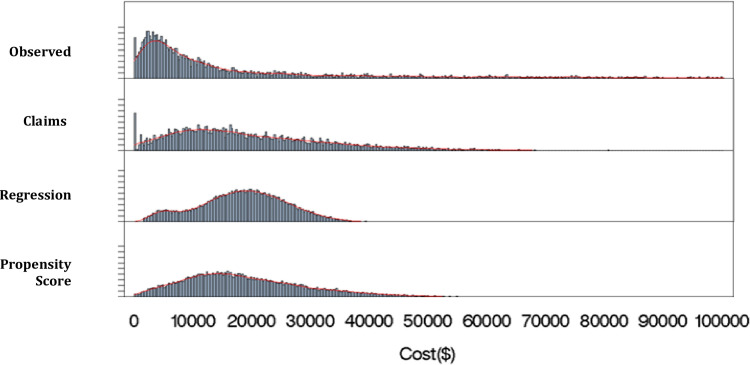
Distribution of observed and predicted spending. The distribution of observed cost was cut off at $100,000, omitting about 2% of beneficiaries. There are no beneficiaries with a predicted spending above $80,500 in any of the models. Here, N = 6,200 and weighted N = 36,824,486. The regression and propensity score methods are based on calibrated claims. The claims approach is based on actual claims except for about 5% of beneficiaries with dollar amounts in the personal summary file(s), no claims for these beneficiaries. For these beneficiaries, we used the imputed claims from the NHANES and assigned dollars to calibrated medical conditions on that basis.

One way to compare the different models is to examine the distribution of spending at the individual level. The distribution of per-person observed spending for the MCBS data set is shown in the first row of Table 2. Mean per capita spending on medical care for the elderly was $17,479 in 2009. The median is much lower: $7,281; as is well known, there is a long right tail in spending, which can be seen in Fig 3 and in the last two columns of Table 2.

**Table 2:** Characteristics of person-level spending from different methods.

		Dollar Amount					N of Beneficiaries with Cost> =	
		(Standard Error)						
	Min	Mean	25%ile	Median	75%ile	Max	$50,000	$100,000
Observed	$0	$17,479 ($389)	$3,291 ($76)	$7,281 ($196)	$17,547 ($525)	$441,857	795	225
Claims-based	$0	$17,479 ($213)	$8,573 ($179)	$15,006 ($271)	$24,230 ($370)	$80,500	153	0
Regression-based	$1,818	$17,479 ($128)	$12,719 ($204)	$17,726 ($157)	$22,496 ($191)	$40,394	0	0
Propensity score-based	$831	$17,479 ($238)	$10,236 ($286)	$15,889 ($246)	$23,202 ($317)	$63,242	36	0

The table shows summary statistics for individual-level spending based on observed costs, costs using the claims-based method, costs using the regression-based method, and costs using the propensity score-based method.

For each attribution method, we estimate spending at the individual level by adding up the cost per disease for all conditions which the person is attributed to have. The second row of Table 2 reports the distribution of the person-level spending from the claims model. By construction, mean person-level spending is the same as in the observed data. Median spending however is significantly higher with the claims-based attribution: $15,006, compared to $7,281 in the observed data. The second panel of Fig 3 shows the distribution of claim-based predicted spending. The right tail of the distribution is substantially compressed. Effectively, the cost of high spending outliers is attributed to relatively common diseases, and these diseases are distributed throughout the population. Thus, the median person spends more but high spenders spend less. As we show, this is a common issue in all the methods we consider.

The third row of Table 2 reports the distribution of the person-level spending from the regression model. The regression approach has less variability in predicted spending than the claims-based approach. For example, the 25^th^ percentile of the predicted spending distribution is 48% higher in the regression approach than the claims-based approach, and the 75^th^ percentile is 7% lower. Effectively, the regression approach has a difficult time determining the cost of different conditions (as typified by the unexplained component), and thus finds less variation in predicted spending across individuals.

The fourth row of Table 2 reports the distribution of the person-level spending from the propensity score model. The propensity score approach shows a broader range of spending than the regression approach but not as broad as the claims-based approach. Effectively, our third stage makes spending at the individual level more dispersed than with the regression but does not allow as many very low and high spenders as with the claims.

A second way to judge the models is using out-of-sample prediction. We estimate the model on a random half of the data and predict spending in the other half, using the same estimation and prediction samples for each model. The out-of-sample root mean square errors (RMSE), shown in Table 3, indicate that the one-part model with log (spending+1) as the dependent variable and with a Gaussian distribution and an identity link has the best fit.

**Table 3:** Characteristics of spending using out-of-sample predictions.

Method	Root Mean Square Error of Prediction
**Claims Based Model**	$28,856
**Regression Models**	
One-part gamma–log link	$42,711
One-part-cubic-root-cost-Gaussian with identity link	$25,228
One-part-log(cost+1)-Gaussian-identity link	$24,410
One-part-Box-Cox model	$26,052
Two-part-gamma-log-link	$42,042
Two-part-cubic-root-cost-Gaussian-identity link	$25,065
Two-part-log(cost+1)–Gaussian-identity	$31,858
**Propensity Score Method**	$22,621

The data are randomly divided into two sub-samples (N = 3,100), one for model fitting and a second for out-of-sample prediction. The Root MSE is for the out-of-sample prediction.

Surprisingly, the Gamma models with a log link perform relatively poorly. These models are sensitive to high-spenders with big residuals–as typified by people in nursing homes. Also surprisingly, two-part models typically used to predict spending do not offer an advantage over one-part models. This is because in our data only 2% of the sample has no spending in the year.

Table 3 shows the out-of-sample RMSE from the propensity score model. The RMSE has a lower standard error than in either the regression model or the claims-based model. Even though we add more parameters, we do not seem to overfit the data.

## Discussion

Our results show many similarities, but also important differences across the alternate methods of assigning costs to medical conditions. The obvious question to ask is, which one is best? There is no gold standard to which the estimates from the different methods can be compared. Thus, we cannot give a definitive answer to the question. Still, some observations are possible.

As noted above, the propensity score model fares the best in out-of-sample prediction of disease costs. A second metric is to compare predicted and actual spending at the individual level. The root of the mean squared difference between actual and predicted spending at the individual level is $2,679 for the claims-based method, $3,032 for the best regression-based method and $2,723 for the propensity score method. We also assessed the correlation between observed and predicted costs at the individual level using each of the three methods. The correlation for the claims score method is 0.63; the correlation for the regression approach is 0.61, and the correlation for the propensity score approach is 0.63.

The ability to implement the models is also important. On this count, the claims-attribution methodology suffers from several problems. First, it is difficult to implement the methodology in a consistent manner. Because most claims–especially the most expensive ones–involve more than one condition, the claims-based methodology will depend on the method used to parcel out costs into conditions. In our sample, 99.5% of dollars involve claims having multiple conditions listed, and 98.4% of beneficiaries have multiple conditions. This makes the claims-based estimation method extremely challenging. We made an assumption about how to divide claims into component conditions, but the assumptions do not have a strong theoretical rationale. Further, the claims-attribution methodology has difficulty with comorbid conditions that are not central to the primary reason for utilization and thus not reliably coded. Relative to the regression and propensity score approaches, the claims-attribution methodology is low for anemia and mental illness, each of which is likely to increase medical spending across-the-board.

The regression and propensity score methods have a lot in common. Both methods facilitate cost attribution to any condition of interest: claim-based, calibrated health condition, self-report, or behavioral risk factors such as smoking. In addition, both methods are designed to adjust for other diseases and demographic covariates. The regression method is easier to implement because it does not require a new model for each health condition. If the data fits the parametric assumptions well and the set of health conditions is not highly correlated, then it produces unbiased and efficient estimates of the attributed costs.

However, the regression-based cost estimation has several limitations. First, it makes several parametric assumptions, which may not be satisfied. Second, there is a large residual spending that cannot be attributed to any disease. Third, some coefficients are estimated to be negative, and the approach we follow assigns zero spending to them. Finally, the variability in spending at the individual level implied by the regression approach is significantly smaller than the actual variation.

Relative to these issues, the propensity score approach has a number of strengths. It requires fewer parametric assumptions and is, therefore, more robust with respect to the high spenders and zero-spenders. Further, the third step of the propensity score approach allows us to account for the number of comorbidities and volume of medical care received. These features of the propensity score method permit us to relax the assumption that health care spending is additive.

In practice, the most important difference between the propensity score method and the regression method is the importance of unattributed spending. There is a base of $58 billion (9% of total spending) that the regression does not attribute to any condition. This is not due to conditions not being included in the model; all diagnoses are included in one of our 78 conditions. Rather, the issue reflects the fact that the regression has a difficult time attributing some spending to conditions and thus leaves it as a constant. In the propensity score model, by contrast, all spending is automatically allocated to conditions.

## Conclusion

While more research is certainly warranted, our tentative conclusion is that the propensity score model offers a good theoretical and empirical methodology to decompose total medical spending to conditions. It predicts disease and individual-level spending at the with the same or better accuracy than other methods, is superior to claims-attribution in handling the many comorbid conditions among the elderly, and avoids the limitations of the regression approach, including negative attributed spending and a large residual cost not assigned to any condition.

## Supporting information

S1 Appendix (DOCX)
