# Supplementary material for: Attributing medical spending to conditions: A comparison of methods
Source: PLoS One. 2020 Aug 10;15(8):e0237082. doi: 10.1371/journal.pone.0237082 (PMC7416958; doi:10.1371/journal.pone.0237082)
Supplement: S1 Appendix — (DOCX) [file pone.0237082.s001.docx]

**S1 Appendix**

In this appendix, we present various technical aspects of the data assembly and analysis.

**A1. Claims-based Method**

In the claims-based method, claims for which there is only one diagnosis are easy to assign to a disease. However, most medical claims have more than one condition associated with them. A typical claim in Medicare data contains a principal diagnosis code (ICD9-CM) and up to 14 secondary diagnoses codes. The question to address is how to estimate the amount spent on treating each of the conditions.

Our version of the claims-based attribution method follows Thorpe’s [1] best guess, making adjustments as appropriate. To start, we note that this method is based on the observed claims for each person, not the calibrated claims – except as noted below. In each set of MCBS claims files (hospital inpatient, hospital outpatient, carrier, hospice, home health, skilled nursing facility and durable medical equipment), we first identify claims with only one listed condition. Table S2 gives detail as to whether each medical condition has claims that satisfy the “single condition claim” criteria. Out of the seven different types of claims, only carrier/physician and hospital outpatient claims have “single condition claims” – that is, a claim with single diagnosis code listed in it – for all medical conditions.

We use this information to apportion a cost to medical conditions for multiple condition claims in these two files. For example, if the average cost of condition ‘a’ in the single condition claim file is c_a_, and the average cost of condition ‘b’ in the single condition claim file is c_b_, the costs of a claim listing both conditions ‘a’ and ‘b’ would be allocated c_a_/(c_a_+c_b_) to condition ‘a’, and c_b_/(c_a_+c_b_) to condition ‘b’.

Unfortunately, the “single-condition claim” criterion fails for inpatient, skilled nursing facility, home health, durable medical equipment, and hospice claims. In these files, only 13-60 conditions satisfy the single condition claim criterion. The paper describes the methodology we use in this case. Using the notation above, the c_a_ and c_b_ are the costs when conditions a and b are the primary reason for the visit. A separate issue is that the prescription drug data in MCBS have no diagnoses codes listed. As described in the text, we assign a DRG weight based on inpatient admissions with that calibrated condition. We then apportion total prescription drug spending for the year based on the share of these DRG weights to each condition.

Finally, for 5.3% of the beneficiaries we find dollar amounts in the personal summary file(s), but we do not see any claims for these beneficiaries. This happens because the event-level files include self-reported events, some of which are not paid under Medicare FFS. For these beneficiaries, we assign dollars based on the calibrated claims instead of actual claims.

**A2. Regression Approach**

The second method of attributing spending to medical conditions is regression analysis. The general equation is given in the text. Following Manning and Mullahy (2001) [2], we explored several GLM estimation techniques: (1) variants of generalized linear models (GLM) for spending with Gamma distribution and a log link function; (2) a GLM model with log(y+1) as the dependent variable with a Gaussian distribution and an identity link; (3) a cubic root model (cubic root of cost); and (4) a Box-Cox model.

Some studies make an additional adjustment for people with no medical spending, for example using a two-part model: [3-4] one equation for the probability of positive spending and the second for the amount spent. We estimate two-part models using a probit to estimate the probability that a beneficiary has positive health care spending. For people with non-zero spending, an OLS or GLM regression is run with the same set of covariates as in the probit model.^5^ We tried several two-part models: (5) generalized linear models (GLM) for spending with a Gamma distribution and a log link function; (6) log(spending) as the dependent variable with a Gaussian distribution and an identity link and finally (7) a cubic root model (cubic root of cost). We could not estimate the two-part Box-Cox model as the STATA software used in the estimation does not support Box-Cox [5].

To implement the regression models, we include as independent variables the 78 calibrated health conditions and screening indicators and other covariates, as described in the text. We combine the estimates from 5 imputed datasets and calculate appropriate standard errors using standard techniques [6,7].

We undertook extensive diagnosis of the residuals from all of the models to choose the most appropriate regression specification, shown in Figures S1-S4. Figure S1 shows the kernel density plot comparison of the residuals (in blue) along with the normal density (in red). Figure S2 shows the standardized normal probability plots of the residuals. Figure S3 shows the quantile of residuals relative to the normal distribution. Figure S4 shows the scatter plot of the residuals relative to per person spending.

Figure S1 shows that the best fitting model is the one-part model with log(spending+1) as the dependent variable and with a Gaussian distribution and an identity link. The one and two part cubic-roots are second best. Surprisingly, the Gamma models with a log link perform relatively poorly. These models are sensitive to high-spenders with big residuals – as typified by people in nursing homes. An alternative way to measure fit is to use out-of-sample predictions.

To transform the non-linear equation to dollars, we use a standard transformation. Our spending model is of the form:

$$log(y_{i}+1)=X_{i}\alpha+D_{i}\beta+\epsilon_{i}$$

where y_i_ is spending during the year, D_i_ is a set of dummy variables for conditions the person has, and X_i_ are other exogenous factors that affect spending, such as age/sex. We estimate the coefficients $\hat{\alpha}$ and $\hat{\beta}$.

To calculate spending on each condition, we start with the fraction of spending attributable to all of the individual’s conditions$(AF_{i})$:

$\hat{AF_{i}}=\frac{\exp\left( A_{i}\hat{\alpha} +D_{i}\hat{\beta} \right)\cdot\hat{S}-\exp\left( A_{i}\hat{\alpha} \right)\cdot\hat{S}}{\exp\left( A_{i}\hat{\alpha} +D_{i}\hat{\beta} \right)\cdot\hat{S}-1}$,

where $\hat{S}=\frac{1}{n}\sum_{i=1}^{n} \exp\left( \hat{\epsilon}_{i} \right)$ is a smearing factor to estimate the average error term. We then calculate the share $\left( M_{ij} \right)$ of total attributable spending by each individual that can be attributed to each diagnosed condition:

$$\hat{M_{ij}}=\frac{\left[ \exp\left( \hat{\beta}_{j} \right)-1 \right]\cdot D_{ij}}{\sum_{j=1}^{J} \left\{ \left[ \exp\left( \hat{\beta}_{j} \right)-1 \right]\cdot D_{ij} \right\}}$$

The observed cost at the individual level is the product of observed cost (*Cost_i_*), the attributable fraction, and the spending share for that condition: $\hat{AC}_{ij}^{\left( T \right)}=\hat{AF}_{i}\cdot\hat{M}_{ij}\cdot{Cost}_{i}$. To form an average cost per condition, we average spending over the beneficiaries with the condition of interest.

**A3. Propensity Score Model**

Our propensity score methodology consists of three steps. The first step divides the population into groups of people with each condition and similar groups without the condition. We start by creating five strata. Creating five strata using propensity score has been shown to be an effective method for applying causal inference to observational studies, removing around 90% of the bias in the difference between the cases and controls caused by covariate imbalance [8].

This process of stratification and estimating the attributable spending is done separately for each of the 78 conditions, using all of the other conditions as covariates. The only exception is that we exclude other medical conditions when they had a deterministic or extremely tight correlation with the condition of the interest; for example people with breast cancer almost never have prostate cancer. Table S5 shows propensity score model exclusions. In general, we use five strata per condition, though conditions with a small number of cases were grouped into fewer strata to avoid situations where a small number of cases has a large impact on the results (see Figure S6). We found good overlap in the covariate profiles between cases and controls to allow us to properly estimate the attributable costs for each condition. Table S6 is the cohort balance table for Acute Myocardial Infarction. We fail to reject equality of the prevalence in almost all cases.

The second step estimates the first-pass cost of each condition as the difference in spending between people with and without each condition.

In the third step, we adjust first-pass spending. There are two reasons for this: first, to ensure that the data match national totals, and second to better match predicted spending at the individual level to actual spending at the individual level. To determine the appropriate adjustments, we compare actual medical expenditures at the individual level to simulated spending based on adding up each individual’s conditions. We relate this to several characteristics such as the number of health conditions, history of hospitalization and institutionalization, and death. Figure S7 plots the difference between first stage and actual cost versus the aforementioned covariates.

Figure S7a relates the spending error to the number of calibrated conditions. When the number of comorbidities is low – roughly 3 or fewer – there is no systematic difference between predicted cost and observed cost: the error is about zero. However, as the number of comorbidities increases, the first stage cost progressively overestimates the observed cost. We have much fewer data for people with a very high number of conditions (30 or more), but the estimates appear to come closer together after that point.

Figure S7b considers whether this pattern is different for people living in the community versus in institutions. Negative residuals are much more prevalent in the institutionalized population, indicating that naïve costs underestimate spending for institutionalized subjects. However, the dependence on number of comorbidities remains similar in both groups.

Figure S7c shows how number of hospitalizations affects costs, given the number of health conditions. The incidence of hospitalization is associated with a higher number of comorbidities. A subgroup of subjects without hospitalizations shows a roughly linear dependence of costs on the number of calibrated conditions. For the subjects who were hospitalized at least for one night, the dependence is nonlinear, and the trend is similar to one observed in Figure S7a. Finally, Figure S7d shows how survivor status affects cost. Death slightly reduces a positive bias of the first stage cost.

We developed an adjustment model that predicts subject-specific observed cost as a product of the sum of the disease costs, ${AC}_{j}$, and a polynomial dependent on the number of the health conditions, history of hospitalization, institutionalization, and death within a given year. The paper describes the equation. Table S3 reports the coefficients. All of the variables are associated with spending in the way we would suggest. All enter as we expect: higher utilization helps close the gap between actual and predicted spending. The cost attribution program for the PSM method is available in our NHA website: <http://www.nber.org/aging/nha/datacreation.html>.

**A4. Disease Calibration**

The disease calibration process is explained more thoroughly in our forthcoming paper Raghunathan et al. (2020) [9]. We have a brief discuss below that we also included in the appendix of our Health Affairs paper (Cutler et al., 2019) [10]. The imputation method proceeds in several steps. We chose to impute the community and institutionalized populations separately given the differences in these populations. We start by appending data from MCBS and respective NHANES. Each person is placed into one of three groups: having the condition in the self-report (NHANES) or claims (MCBS); not having the condition if the NHANES self-report indicates the subject does not have health condition and there is no claim for the condition; and missing if there is no claim for the health condition in the MCBS. We now have a standard missing data problem for which we use sequential regression multivariate imputation procedure.

For conditions present in NHANES, let D(−j) denote the collection of disease indicators for all diseases except disease j. We construct a propensity score for having disease j based on fitting a logistic regression model to the other conditions and exogenous covariates, X, and predicting with (X, D(−j)) strata based on the propensity scores. Covariates included age, gender, education level, military service, race/ethnicity, marital status, current smoking, ever smoking, pneumonia shot, flu shot, hysterectomy, poverty level, private health insurance, difficulty lifting/carrying 10 pounds, difficulty stooping/crouching/kneeling, difficulty walking quarter mile, difficulty dressing, difficulty eating, general self-rated health status, self-rated health compared to 1 year ago, use of hearing aid, institutional days, inpatient hospital nights, inpatient hospital stays, height, weight, and probability of death.

Within each propensity score classes, we estimate the prevalence rate using the self-report, S_j_, and the claims C_j_. If the prevalence rate based on the claims is greater than or equal to that based on the self-report, then we set all missing D_j_ to 0. That is, no additional imputation is necessary and all those with no claims are considered as not having that health condition. If the self-report prevalence rate is greater than the prevalence rate based on the claims, we randomly set some missing D_j_ to 1 so that the prevalence rates after the imputation match the self-report prevalence rates. We used five Bernoulli draws within each propensity score class to achieve this calibration. Thus, we have five imputed data sets.

Note that medical expenditure and health conditions without self-report are missing in the NHANES portion of the appended data. To be fully conditional, these missing values were imputed in the NHANES. These two steps – the disease imputations into MCBS and the medical spending/health condition imputations into NHANES – were iterated across all diseases several times until the multiply imputed prevalence rates stabilized.

The regression relationship between the multiply imputed D_j_ and claims based C_j_ for conditions available in NHANES may be viewed as a measurement error model and this relationship is then used to calibrate other health conditions not present in NHANES. In this step, we chose the most similar prevalent condition for the imputation.

The NHANES is a sample of the community dwelling population only. Thus, the claims imputation for the institutionalized sample required some differences. For this population, the calibrated non-institutionalized MCBS data was considered as the ‘donor’ survey in imputing condition prevalence in the institutionalized population. For each claim, subjects were matched according to the estimated propensity of being institutionalized given the self-report and demographic information, and the remaining claims. To estimate this propensity, logistic regression was utilized with a forward selection procedure on the principal components of the set of variables of interest. Principal component analysis was used in an effort to explain as much of the variation in propensity scores as possible while avoiding a complete separation of data points given the small number of people who are institutionalized. Assuming that the probability of being calibrated is the same conditional on institutionalization status, calibrated conditions are drawn for the institutionalized population matching the distribution for the community population.

The calibration process produces five imputed data sets for both community and institutionalized population. We use all five imputed data sets in our analysis using appropriate survey weight and sample design adjustments. Importantly, prevalence based on the calibrated conditions indicates diagnosis or treatment either currently or any time in the past. We denote these prevalence rates P_j_ for condition j.

**References**

1. Thorpe KE, Florence CS, Joski P. Which medical conditions account for the rise in health care spending? Health Affairs. 2004; 23: w437-w445.

2. Manning WG, Mullahy J. Estimating log models: to transform or not to transform? Journal of Health Economics. 2001; 20(4): 461-494.

3. Duan, N, Manning WG Jr, Morris CN, Newhouse JP. Choosing between the sample-selection model and the multi-part model. Journal of Business and Economic Statistics. 1984; 2: 283-289.

4. Mihaylova B, Briggs A, O’Hagan A, Thompson SG. Review of statistical methods for analyzing healthcare resources and costs. Health Economics. 2011; 20: 897-916.

5. Belotti F, Deb P, Manning WG, Norton EC. twopm: Two-part models. *Stata Journal*. 2015; 15(1): 3-20.

6. Little R, and Rubin D, *Statistical Analysis with Missing Data*. New York: John Wiley & Sons, 1987.

7. Li K, Raghunathan T, and Rubin D, “Large sample significance levels from multiply-imputed data using moment-based statistics and an F reference distribution,” *Journal of the American Statistical Association*, 1991; 86: 1065–1073.

8. Rosenbaum, Paul R., and Donald B. Rubin. 1984. Reducing bias in observational studies using subclassification on the propensity score. *Journal of the American Statistical Association* 79(387): 516-524.

9. Raghunathan T, et al., “Combining information from multiple data sources to assess population health”, *Journal of Survey Statistics and Methodology*, (2020-forthcoming).

10. David M. Cutler, Kaushik Ghosh, Kassandra L. Messer, Trivellore E. Raghunathan, Susan T. Stewart, and Allison B. Rosen. “Explaining The Slowdown In Medical Spending Growth Among The Elderly, 1999–2012” , Health Affairs, Vol. 38, No. 2, 2019

**Figure S1: Kernel density plot of residuals from regression models**

**Figure S2: Standardized normal probability plot of residuals**

**Figure S3: The quantile of residual against the quintiles of the normal distribution**

**Figure S4: Scatter plot of Residuals**

**Acute Renal Failure Deep Vein Thrombosis**

**
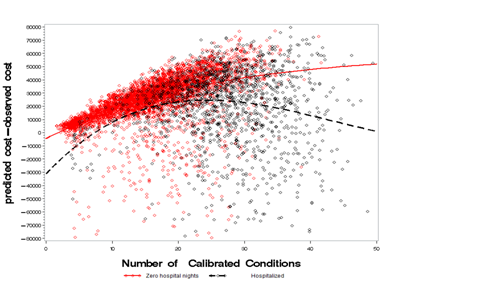

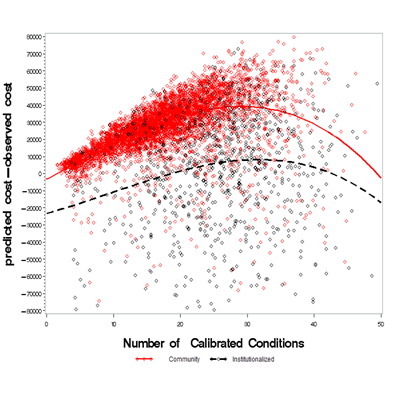

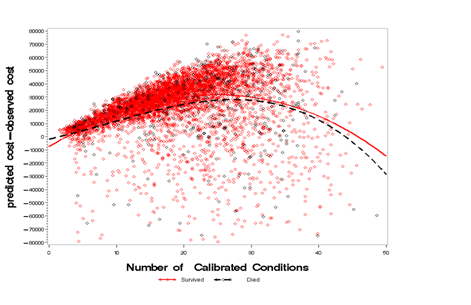

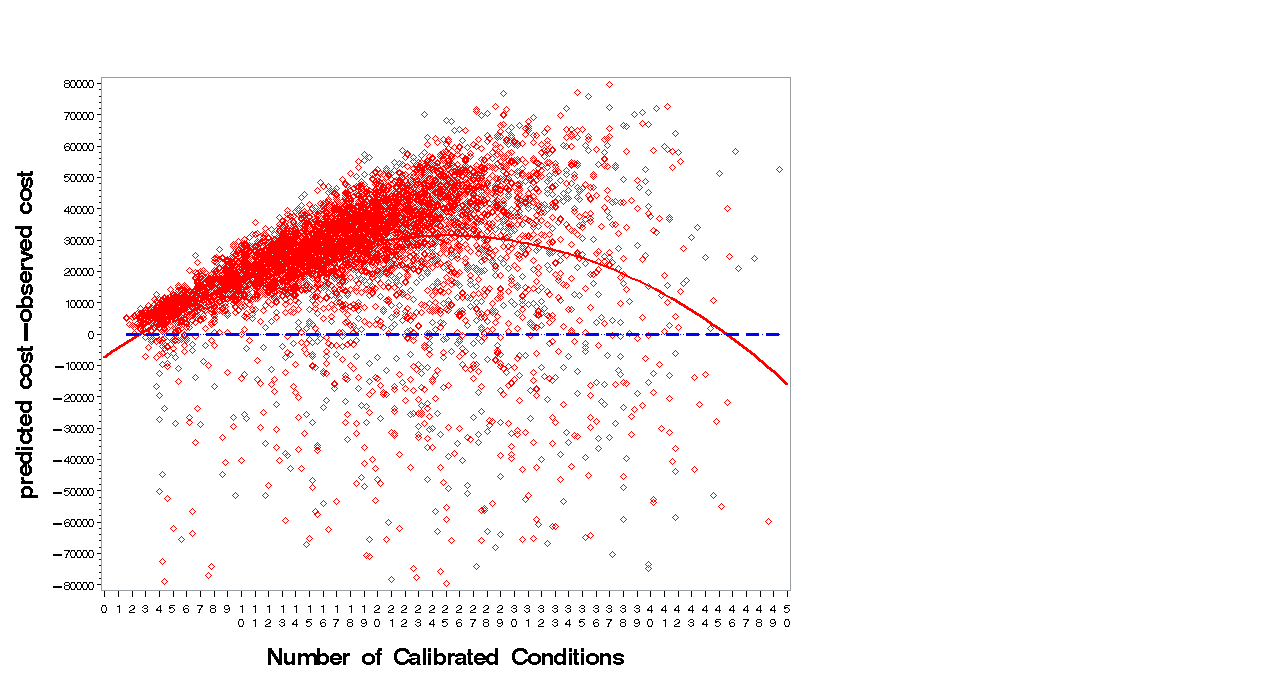
Figure S7: Predicted vs. Observed Cost**

**S7d**

**S7c**

**S7b**

**S7a**

**Note:** Figures S7a – S7d show the error term, measured as the difference between naïve predicted cost and true cost against the number of health conditions, history of hospitalization and institutionalization and death. The results suggest a need for the non-linear adjustment.

**Table S1: Conditions in each of the multiple CCS categories and prevalence rates**

| **Multi-level CCS / Condition label (ID)** | **Prevalence (Calibrated Conditions)** | **Combined Prevalence**  **(Multi-level CCS)** |
| --- | --- | --- |
| **1-Infectious and parasitic diseases** |  | **65%** |
| Immunizations and screening for infectious disease (4) | 53% |  |
| Tuberculosis, Other Infectious disease (106) | 28% |  |
| **2-Neoplasms** |  | **50%** |
| Cervical Cancer and Other Cancer (120) | 22% |  |
| Benign Neoplasm (13) | 22% |  |
| Skin Cancer (8) | 14% |  |
| Breast Cancer (9) | 6% |  |
| Prostate Cancer (11) | 6% |  |
| Colon Cancer (6) | 3% |  |
| Lung Cancer (7) | 2% |  |
| Hematologic Cancers (12) | 2% |  |
| **3-Endocrine; nutritional; and metabolic diseases and immunity disorders** |  | **83%** |
| Hyperlipidemia (18) | 62% |  |
| Other Endocrine Diseases (21) | 38% |  |
| Diabetes Mellitus (16) | 32% |  |
| Thyroid Disorders (15) | 28% |  |
| Gout and other crystal arthropathies (20) | 8% |  |
| Undiagnosed Diabetes Mellitus (17) | 2% |  |
| Undiagnosed Hyperlipidemia (19) | 2% |  |
| **4-Diseases of the blood and blood-forming organs** |  | **35%** |
| Anemias (22) | 29% |  |
| Other Hematologic Disease (23) | 12% |  |
| **5-Mental Illness** |  | **46%** |
| ETOH Abuse, Illicit Drug Use, Tobacco Use (107) | 13% |  |
| Depression (28) | 13% |  |
| Anxiety and Posttraumatic Stress Disorder (108) | 13% |  |
| Dementia (27) | 12% |  |
| ADD-ADHD, Other Mental Health Disorders (109) | 10% |  |
| Schizophrenia (30) | 5% |  |
| Bipolar disorder (29) | 3% |  |

|  |  |  |
| --- | --- | --- |

**Table S1 (Contd.): Conditions in each of the multiple CCS categories and prevalence rates**

| **Multi-level CCS /Condition label (ID)** | **Prevalence (Calibrated Conditions)** | **Combined Prevalence**  **(Multi-level CCS)** |
| --- | --- | --- |
| **6-Diseases of the nervous system and sense organs** |  | **82%** |
| Eye Disorders (45) | 43% |  |
| Cataract (43) | 40% |  |
| Other Disease of the Central Nervous System (48) | 31% |  |
| Glaucoma (44) | 20% |  |
| Other Ear Disorders (47) | 16% |  |
| Vestibular Disorders (46) | 14% |  |
| Headaches and Migraine (111) | 12% |  |
| Parkinson's Disease, Multiple Sclerosis, Paralysis (110) | 9% |  |
| Seizure Disorders (40) | 6% |  |
| Otitis Media (36) | 6% |  |
| **7-Diseases of the circulatory system** |  | **90%** |
| Hypertension (49) | 71% |  |
| Other Vascular Diseases (62) | 36% |  |
| Atrial Fibrillation and Flutter, Other Arrhythmias (112) | 32% |  |
| Other Cardiovascular Diseases (61) | 31% |  |
| Coronary Atherosclerosis and other heart disease (52) | 27% |  |
| Peripheral Vascular Disease (60) | 20% |  |
| Cerebrovascular disease (59) | 18% |  |
| Congestive Heart Failure (56) | 18% |  |
| Acute myocardial infarction (51) | 11% |  |
| Acute hemorrhagic stroke, Acute ischemic stroke (113) | 9% |  |
| Deep Vein Thrombosis or DVT (64) | 6% |  |
| Pulmonary Embolism (63) | 4% |  |
| Undiagnosed Hypertension (50) | 2% |  |
| Cardiac Arrest (55) | 3% |  |
| **8-Diseases of the respiratory system** |  | **66%** |
| Respiratory symptoms (70) | 38% |  |
| Acute respiratory infection (69) | 27% |  |
| Other Respiratory Diseases (71) | 26% |  |
| Chronic Obstructive Pulmonary Disease (67) | 18% |  |
| Asthma (68) | 14% |  |
| Pneumonia (65) | 11% |  |
| Influenza (66) | 3% |  |
|  |  |  |

**Table S1 (Contd.): Conditions in each of the multiple CCS categories and prevalence rates**

| **Multi-level CCS /Condition label (ID)** | **Prevalence (Calibrated Conditions)** | **Combined Prevalence**  **(Multi-level CCS)** |
| --- | --- | --- |
| **9-Diseases of the digestive system** |  | **57%** |
| Reflux/Ulcer Disease, Biliary Tract Disease, Liver Disease, Gastrointestinal Bleeding, Other Gastrointestinal Disorders (114) | 57% |  |
| **10-Diseases of the genitourinary system** |  | **64%** |
| UTI, Urinary Incontinence, Other Genitourinary Diseases (116) | 54% |  |
| Acute Renal Failure (77) | 9% |  |
| Chronic Renal Failure, End-stage Renal Disease (ESRD) (115) | 15% |  |
| Hyperplasia of the Prostate (82) | 15% |  |
| **11-Complications of menopause; pregnancy; childbirth** |  | **10%** |
| Menopause, Pregnancy and Childbirth, Contraception and Procreation (117) | 10% |  |
| **12-Diseases of the skin and subcutaneous tissue** |  | **42%** |
| Dermatologic Diseases (87) | 42% |  |
| **13-Diseases of the musculoskeletal system and connective tissue** |  | **82%** |
| Other Rheumatic Diseases (92) | 58% |  |
| Osteoarthritis (89) | 49% |  |
| Back Pain (90) | 34% |  |
| Osteoporosis (91) | 18% |  |
| Rheumatoid Arthritis (88) | 9% |  |
| **14-Congenital anomalies & Certain conditions originating in the perinatal period** |  | **10%** |
|  |  |  |
| Congenital Disorders, Newborn conditions (118) | 10% |  |
| **15-Injury and poisoning** |  | **47%** |
| Trauma, Fractures, Traumas, Poisoning, Motor vehicle accident (119) |  |  |
|  | 46% |  |
| Hip Fracture (96) | 4% |  |

**Table S1 (Contd.): Conditions in each multiple CCS categories and prevalence rates**

| **Multi-level CCS /Condition label (ID)** | **Prevalence (Calibrated Conditions)** | **Combined Prevalence**  **(Multi-level CCS)** |
| --- | --- | --- |
| **16-Other conditions** |  | **83%** |
| Signs and Symptoms (99) | 74% |  |
| Residual, unclassified, all other E codes (101) | 48% |  |
| **17-Screening** |  | **41%** |
| Screening: Breast Cancer (102) | 22% |  |
| Screening: Prostate Cancer (104) | 13% |  |
| Screening: Cervical Cancer (105) | 9% |  |
| Screening: Colon Cancer (103) | 8% |  |

**Note:** Prevalence rates are from calibrated health conditions. We use the information from NHANES to compare, calibrate and refine the estimates of medical conditions in MCBS. First, missing data on demographic, socio-economic and self-reported medical conditions in MCBS and NHANES are imputed using a sequential regression multiple–imputation procedure. We then use three types of calibration processes to impute a set of 101 health indicators and 4 screening variables that we call “calibrated medical conditions.” These three types of calibration process include: (1) calibration of conditions that have self-report available in NHANES; (2) calibration of conditions with no self-report in NHANES and (3) calibration of the institutionalized population for all conditions. This calibration process produces five imputed data sets. The average prevalence rates reported here are calculated using all five imputed data sets using MIANALYZE (in SAS^[[1]](#footnote-1)^). Table S1 is sorted from highest to lowest prevalence within each broad disease category.

| **Table S2: Percentage of conditions with claims reporting single conditions in MCBS** | |
| --- | --- |
| **File** | **Number** |
| Carrier | 100% |
| Outpatient | 100% |
| Inpatient | 13% |
| Skilled Nursing Facility | 35% |
| Home Health Agency | 20% |
| Durable Medical Equipment | 58% |
| Hospice | 39% |
| Prescription Drug | N/A |
| **Note:** The sample is the 105 conditions obtained after collapsing and expanding the CCS codes, less the conditions of undiagnosed hypertension, high cholesterol, and diabetes. N/A=Not Applicable (ICD9-CM diagnosis codes in Prescription Drugs claims not available) | |

**Table S3: Adjusted costs model estimates (propensity score method)**

| **Parameters** | **Coefficient**  **(Standard Error)** |
| --- | --- |
| Intercept | 0.3956 (0.0462) |
| Number of comorbidities | -0.0042 (0.0015) |
| Number of comorbidities squared | 0.0001 (0.0001) |
| Any hospitalization | -0.1663 (0.0166) |
| Number of nights in hospital | 0.0143 (0.0004) |
| Number of hospital admissions | 0.0246 (0.0068) |
| Number of days in an institution | 0.0021 (0.0000) |
| Patient survived the calendar year | 0.0828 (0.0332) |
| Number of months survived in the calendar year  (if deceased) | 0.0071 (0.0042) |
| Number of outpatient claims | 0.0058 (0.0007) |

**Note:** The N in each imputed dataset is 6,200. The adjusted R^2^ is 0.68.

**Table S4: Costs attributed to medical conditions using different methods**

|  | |  | **Average Cost** | | | **Totals Cost (in Billions)** | | |
| --- | --- | --- | --- | --- | --- | --- | --- | --- |
| **Multiple-CCS/Conditions** | | | **Claims** | **Regression** | **Propensity Score** | **Claims** | **Regression** | **Propensity Score** |
| **1-Infectious and parasitic diseases** | | |  | | |  | | |
|  | Immunizations and screening for infectious disease | | $22 | $556 | $15 | $0.40 | $10.89 | $0.30 |
|  | Tuberculosis, STD, non-HIV, HIV, Other Infectious disease | |  |  |  |  |  |  |
|  |  |  | 2,469 | 163 | 1,372 | 19.88 | 1.68 | 14.14 |
| **2-Neoplasms** | | |  | | |  | | |
|  | Colon cancer | | 2,802 | 807 | 1,240 | 1.89 | 0.92 | 1.41 |
|  | Lung Cancer | | 4,102 | 2,758 | 4,444 | 2.30 | 1.56 | 2.52 |
|  | Skin Cancer | | 1,547 | 126 | 308 | 5.17 | 0.65 | 1.58 |
|  | Breast Cancer | | 1,737 | 1,286 | 1,123 | 2.86 | 2.59 | 2.28 |
|  | Prostate Cancer | | 1,832 | 1,671 | 988 | 3.58 | 3.43 | 2.05 |
|  | Hematologic Cancers | | 4,214 | 2,131 | 3,431 | 2.60 | 1.73 | 2.82 |
|  | Benign Neoplasm | | 1,309 | 710 | 47 | 8.04 | 5.76 | 0.38 |
|  | Cervical Cancer and Other Cancers | | 2,410 | 1,317 | 1,143 | 15.00 | 10.90 | 9.46 |
| **3-Endocrine; nutritional; and metabolic diseases**  **and immunity disorders** | | |  | | |  | | |
|  | Thyroid Disorders | | 859 | 322 | 448 | 7.10 | 3.29 | 4.59 |
|  | Diabetes Mellitus | | 862 | 1,454 | 1,119 | 10.19 | 17.22 | 13.25 |
|  | Undiagnosed Diabetes Mellitus | | 637 | 485 | 43 | 0.01 | 0.33 | 0.03 |
|  | Hyperlipidemia | | 623 | 806 | 720 | 14.04 | 18.51 | 16.57 |
|  | Undiagnosed Hyperlipidemia | | 540 | 445 | -856 | 0.07 | 0.25 | -0.51 |
|  | Gout and other crystal arthropathies | | 825 | 119 | 384 | 1.28 | 0.34 | 1.11 |
|  | Other Endocrine Diseases | | 1,362 | 639 | 1,422 | 16.47 | 8.92 | 19.81 |

**Table S4 (Contd.): Costs attributed to medical conditions using different methods**

|  | |  | **Average Cost** | | | **Totals Cost (in Billions)** | | | |
| --- | --- | --- | --- | --- | --- | --- | --- | --- | --- |
|  | **Multiple-CCS/ Conditions** | | **Claims** | **Regression** | **Propensity Score** | **Claims** | | **Regression** | **Propensity Score** |
| **4-Diseases of the blood and blood-forming organs** | | |  | | | |  | | |
|  | Anemia | | $1,393 | $948 | $2,147 | $12.22 | | $9.99 | $22.62 |
|  | Other Hematologic Disease | | 1,818 | 662 | 2,328 | 5.44 | | 2.87 | 10.15 |
| **5-Mental Illness** | | |  | | | |  | | |
|  | ETOH Abuse, Illicit Drug and Tobacco | | 2,156 | 1,275 | 1,188 | 5.77 | | 6.22 | 5.85 |
|  | Dementia | | 3,388 | 783 | 2,229 | 10.93 | | 3.53 | 10.11 |
|  | Depression | | 1,724 | 815 | 1,812 | 5.85 | | 3.90 | 8.65 |
|  | Bipolar Disorder | | 1,451 | 405 | 654 | 0.34 | | 0.43 | 0.62 |
|  | Schizophrenia | | 1,850 | 909 | 3,522 | 1.81 | | 1.78 | 6.94 |
|  | Anxiety, Posttraumatic Stress Disorder | | 1,017 | 254 | 450 | 3.01 | | 1.22 | 2.20 |
|  | Attention Deficit Hyperactivity Disorder ADD-ADHD., Mental Retardation (HCC term), Other Mental Health Disorders | |  |  |  |  | |  |  |
|  |  |  | 1,526 | 346 | 1,429 | 3.05 | | 1.25 | 5.12 |
| **6-Diseases of the nervous system and sense organs** | | |  | | | |  | | |
|  | Otitis Media | | 673 | 284 | 379 | 0.77 | | 0.68 | 0.90 |
|  | Vestibular Disorders | | 699 | 40 | 351 | 2.58 | | 0.21 | 1.80 |
|  | Other Ear Disorders | | 854 | 461 | 165 | 3.77 | | 2.78 | 1.00 |
|  | Parkinson's Disease, MS, Paralysis | | 2,638 | 849 | 1,635 | 3.29 | | 2.88 | 5.48 |
|  | Seizure Disorders | | 1,893 | 549 | 1,984 | 1.61 | | 1.16 | 4.07 |
|  | Headaches, Migraine | | 753 | 199 | 169 | 1.85 | | 0.93 | 0.77 |
|  | Cataract | | 960 | 490 | 146 | 12.40 | | 7.21 | 2.15 |
|  | Glaucoma | | 701 | 544 | 141 | 3.70 | | 3.95 | 1.02 |
|  | Eye Disorders | | 1,017 | 615 | 174 | 14.25 | | 9.69 | 2.75 |
|  | Disease of the Central Nervous System | | 2,089 | 1,192 | 2,203 | 19.76 | | 13.53 | 24.99 |

**Table S4 (Contd.): Costs attributed to medical conditions using different methods**

|  | |  | | **Average Cost** | | | **Total Cost (in Billions)** | | |
| --- | --- | --- | --- | --- | --- | --- | --- | --- | --- |
|  | **Multiple-CCS/Calibrated Conditions** | |  | **Claims** | **Regression** | **Propensity Score** | **Claims** | **Regression** | **Propensity Score** |
| **7-Diseases of the circulatory system** | | |  |  |  |  |  |  |  |
|  | Hypertension | |  | $883 | $2,619 | $1,347 | $22.33 | $68.86 | $35.42 |
|  | Undiagnosed Hypertension | |  | 840 | 445 | -897 | 0.18 | 0.37 | -0.81 |
|  | Acute myocardial infarction (AMI) | |  | 4,680 | 459 | 1,142 | 4.63 | 1.98 | 4.71 |
|  | Coronary Athero. and other heart disease | |  | 2,524 | 1,371 | 1,687 | 24.64 | 13.40 | 16.49 |
|  | Atrial Fib. and Flutter, Other Arrhythmias | |  | 1,293 | 486 | 1,114 | 11.41 | 5.63 | 12.97 |
|  | Cardiac Arrest (includes VF) | |  | 4,407 | 1,384 | 2,400 | 1.64 | 1.50 | 2.63 |
|  | Congestive Heart Failure | |  | 2,009 | 375 | 2,514 | 10.18 | 2.41 | 16.20 |
|  | Acute hemorrhagic or Ischemic Stroke | |  | 2,473 | 452 | 1,593 | 5.09 | 1.46 | 5.06 |
|  | Cerebrovascular Disease | |  | 1,470 | 514 | 1,341 | 7.60 | 3.46 | 9.01 |
|  | Peripheral Vascular Disease | |  | 1,873 | 158 | 1,071 | 10.42 | 1.15 | 7.75 |
|  | Other Cardiovascular Diseases | |  | 2,692 | 971 | 1,346 | 25.66 | 11.05 | 15.32 |
|  | Other Vascular Diseases | |  | 1,848 | 552 | 1,137 | 20.81 | 7.30 | 15.07 |
|  | Pulmonary embolism | |  | 2,993 | 486 | 1,593 | 1.30 | 0.70 | 2.31 |
|  | Deep Vein Thrombosis (DVT) | |  | 2,259 | ----- | 1,200 | 2.22 | ----- | 2.68 |
| **8-Diseases of the respiratory system** | | |  |  |  |  |  |  |  |
|  | Pneumonia (non-TB, non-STD) | |  | 2,909 | 285 | 2,318 | 8.01 | 1.14 | 9.31 |
|  | Influenza | |  | 958 | 897 | 170 | 0.29 | 1.13 | 0.21 |
|  | Chronic Obstructive Pulmonary Disease | |  | 1,373 | 1,079 | 1,477 | 9.16 | 7.22 | 9.88 |
|  | Asthma | |  | 1,178 | 411 | 817 | 3.37 | 1.99 | 4.07 |
|  | Acute respiratory infection | |  | 604 | 331 | 83 | 4.67 | 3.29 | 0.82 |
|  | Respiratory symptoms | |  | 1,033 | 901 | 1,669 | 12.39 | 12.45 | 23.05 |
|  | Other Respiratory Diseases | |  | 2,073 | 877 | 1,592 | 15.55 | 8.27 | 15.05 |

**Table S4 (Contd.): Costs attributed to medical conditions using different methods**

|  | |  | **Average Cost** | | | **Total Cost (in billions)** | | |
| --- | --- | --- | --- | --- | --- | --- | --- | --- |
|  | **Multiple-CCS/Calibrated Conditions** | | **Claims** | **Regression** | **Propensity Score** | **Claims** | **Regression** | **Propensity Score** |
| **9-Diseases of the digestive system** | | |  | | |  | | |
|  | Reflux/Ulcer Disease, Biliary Tract Disease, Liver Disease, Gastrointestinal Bleeding, Other Gastrointestinal Disorders | |  |  |  |  |  |  |
|  |  |  | $1,747 | $1,588 | $1,370 | $28.06 | $33.09 | $28.52 |
| **10-Diseases of the genitourinary system** | | |  | | |  | | |
|  | Acute Renal Failure | | 3,738 | ---- | 3,080 | 7.74 | ----- | 10.65 |
|  | Chronic Renal Failure & ESRD | | 2,449 | 601 | 1,757 | 8.39 | 3.32 | 9.74 |
|  | UTI, Urinary Incontinence, Other Genitourinary Diseases | | 1,096 | 891 | 1,020 | 18.12 | 17.60 | 20.14 |
|  | Hyperplasia of the Prostate | | 813 | 1,369 | 187 | 3.85 | 7.65 | 1.05 |
| **11-Complications of menopause, pregnancy, childbirth, and the puerperium** | | |  | | |  | | |
|  | Menopause, Pregnancy and Childbirth, Contraception and Procreation | | 563 | 130 | -45 | 1.43 | 0.46 | -0.16 |
|  |  |  |  |  |  |  |  |  |
| **12-Diseases of the skin and**  **subcutaneous tissue** | | |  | | |  | | |
|  | Dermatologic Diseases | | 912 | 817 | 509 | 12.61 | 12.70 | 7.91 |
| **13-Diseases of the musculoskeletal system**  **and connective tissue** | | |  | | |  | | |
|  | Rheumatoid Arthritis | | 2,130 | 399 | 843 | 2.66 | 1.26 | 2.71 |
|  | Osteoarthritis | | 1,774 | 66 | 277 | 17.96 | 1.22 | 5.05 |
|  | Back Pain | | 1,260 | 601 | 349 | 13.32 | 7.58 | 4.40 |
|  | Osteoporosis | | 1,053 | 236 | 307 | 5.11 | 1.57 | 2.08 |
|  | Other Rheumatic Diseases | | 1,149 | 1,406 | 1,024 | 23.14 | 29.98 | 21.83 |

**Table S4 (Contd.): Costs attributed to medical conditions using different methods**

|  | |  | | **Average Cost** | | | **Totals Cost (in Billions)** | | |
| --- | --- | --- | --- | --- | --- | --- | --- | --- | --- |
|  | **Multiple-CCS/Calibrated Conditions** | |  | **Claims** | **Regression** | **Propensity Score** | **Claims** | **Regression** | **Propensity Score** |
| **14 -Congenital Anomalies & Certain**  **conditions originating in the perinatal period** | | |  | | | |  | | |
|  | Congenital Disorders, Newborn conditions | |  | $2,237 | $654 | 1,272 | $4.63 | $2.38 | $4.64 |
| **15-Injury and poisoning** | | |  | | | |  | | |
|  | Trauma, Fractures, Poisoning and other injury, Motor vehicle accident | |  |  |  |  |  |  |  |
|  |  |  |  | 2,033 | 1,520 | 2,163 | 25.83 | 25.46 | 36.25 |
|  | Hip Fracture | |  | 3,293 | 425 | 456 | 1.63 | 0.66 | 0.73 |
| **16-Other conditions** | | |  | | | |  | | |
|  | Signs and Symptoms | |  | 829 | 3,747 | 2,305 | 22.13 | 102.37 | 62.97 |
|  | Residual, unclassified, all other E codes | |  | 1,182 | 1,051 | 1,146 | 19.20 | 18.76 | 20.46 |
| **17-Cancer Screening** | | |  | | | |  | | |
|  | Screening: Breast Cancer | |  | 243 | 61 | -350 | 1.79 | 0.50 | -2.89 |
|  | Screening: Colon Cancer | |  | 231 | 177 | -561 | 0.67 | 0.52 | -1.67 |
|  | Screening: Prostate Cancer | |  | 90 | 157 | -945 | 0.34 | 0.77 | -4.67 |
|  | Screening: Cervical Cancer | |  | 103 | 41 | -691 | 0.21 | 0.13 | -2.23 |
|  | Other covariates (including intercept) | |  | N/A | 1,566 | N/A | N/A | 57.68 | N/A |

**Note:** Cost attribution in the “claims based approach” uses health conditions reported in 2009 MCBS. Cost attribution in regression and propensity score methods uses calibrated health conditions (refer to data section for details on calibrated health conditions). Regression coefficients are negative in all five imputed data sets for Acute Renal Failure and Deep Vein Thrombosis (DVT). No spending is attributed to these conditions in regression-based approach. N/A = Not applicable.

|  | | |
| --- | --- | --- |
| **Conditions** | **Conditions/covariates** | |
| [1] Congestive Heart Failure | | Atrial Fibrillation and Flutter, Other Arrhythmias |
| [2] Cardiac Arrest (includes VF) | | Atrial Fibrillation and Flutter, Other Arrhythmias |
| [3] Other Cardiovascular Diseases | | Coronary Atherosclerosis and other heart disease |
| [4] Diabetes Mellitus | | Undiagnosed Diabetes/Hypertension/Hyperlipidemia |
| [5] Hyperlipidemia | | Undiagnosed Diabetes/Hypertension/Hyperlipidemia |
| [6] Hypertension | | Undiagnosed Diabetes/Hypertension/Hyperlipidemia |
| [7] Lung Cancer | | Ever smoke, Smoke now |
| [8] Prostate Cancer | | Male, had hysterectomy, pap smear, mammogram, Breast Cancer, Cervical Cancer, Pregnancy and Childbirth, Menopause, Contraception and Procreation, Breast Cancer Screening, Cervical Cancer Screening, Cancer, Reproductive (female) |
| [9] Breast Cancer | | Male, PSA test in last year, Prostate Cancer, Hyperplasia of the Prostate, Screening: Prostate Cancer, Screening: Breast Cancer |
| [10] Anxiety/PTSD | | ETOH Abuse, Illicit Drug Use, Tobacco Use |
| [11] Cataract | | Eye Disorders, Glaucoma |
| [12] Glaucoma | | Cataract, Eye Disorders |
| [13] Eye Disorders | | Cataract, Glaucoma |
| [14] Reproductive(female) | | Male, Prostate Cancer, Hyperplasia of the Prostate |
| [15] Acute respiratory infection | | Respiratory symptoms, Other Respiratory Diseases |
| [16] Respiratory symptoms | | COPD, Acute respiratory infection, Other Respiratory Diseases |
| [17] Other Respiratory Diseases | | Chronic Obstructive Pulmonary Disease, Acute respiratory infection, Other Respiratory Diseases |
| [18] COPD | | Asthma |
| [19] Asthma | | COPD, Acute respiratory infection, Respiratory symptoms, Other Respiratory Diseases |
| [20] Pneumonia | | Pneumonia vaccination, Influenza, Immunizations and screening for infectious disease, Tuberculosis, STD, non-HIV, HIV, Other Infectious disease |
| [21] Influenza | | Flu shot last year |
| [22] Infectious disease | | Flu shot last year, Pneumonia vaccination (ever had) |
| [23] Acute Renal Failure | | Chronic Renal failure |
| [24] Hyperplasia of the Prostate | | Male, had hysterectomy, pap smear, mammogram, Breast Cancer, Cervical Cancer, Pregnancy and Childbirth, Menopause, Contraception and Procreation, Screening: Breast Cancer, Screening: Cervical Cancer, Reproductive(female) |
| [25] Hip Fracture | | Difficulty walking, Difficulty stooping |
| [26] Accidents and Ecodes | | Generic Illness |
| [27] Osteoarthritis | | Other Rheumatism Diseases |
| [28] Signs and Symptoms | | Residual, unclassified, all other E codes, Trauma, etc. Motor vehicle accident |
| [29] Screening: Breast Cancer | | Male, PSA test in last year, Prostate Cancer, Hyperplasia of the Prostate, Screening: Prostate Cancer |
| [30] Screening: Colon Cancer | | Colon Cancer |
| [31] Screening: Prostate Cancer | | Male, had hysterectomy, pap smear, mammogram, Prostate Cancer, Hyperplasia of the Prostate |
| [32] Screening: Cervical Cancer | | Male, pap smear, Other Cancer, PSA test in last year, Screening: Prostate Cancer |

**Table S5: Propensity score models exclusions**

| **Variables**^1^ | **Yes** | **No** | **p-value** |
| --- | --- | --- | --- |
| Age (continuous) | 77.52 | 77.37 | 0.37 |
| Male | 40% | 40% | 0.94 |
| Education (9^th^-12^th^ (no diploma)) | 17% | 17% | 0.76 |
| Education (high school diploma) | 28% | 28% | 0.9 |
| Education (associate degree/some college) | 20% | 20% | 0.92 |
| Education (college grad and above) | 14% | 14% | 0.92 |
| Military service (ever served in Armed Forces) | 25% | 26% | 0.65 |
| Race (non-Hispanic black) | 7% | 8% | 0.32 |
| Race (Hispanic) | 5% | 5% | 0.5 |
| Race (Other) | 3% | 3% | 0.77 |
| Marital status (widowed) | 41% | 41% | 0.76 |
| Marital status (divorced/separated) | 6% | 6% | 0.94 |
| Marital status (never married) | 4% | 4% | 0.77 |
| Smoke now | 9% | 10% | 0.42 |
| Ever smoke | 54% | 54% | 0.93 |
| Pneumonia shot (ever had a shot for pneumonia) | 66% | 65% | 0.37 |
| Flu shot (had a seasonal flu shot for last winter) | 72% | 71% | 0.62 |
| Hysterectomy (ever had hysterectomy) | 25% | 25% | 0.88 |
| Poverty level: 100-125% of FPL^2^ | 10% | 10% | 0.86 |
| Poverty level: 125-200% of FPL | 20% | 20% | 0.84 |
| Poverty level: 200-400% of FPL | 28% | 28% | 0.73 |
| Poverty level: >400% of FPL | 15% | 15% | 0.92 |
| Other private health insurance during year | 74% | 73% | 0.31 |
| Difficulty lifting (10 lbs, scale 1-4^3^) | 1.78 | 1.75 | 0.15 |
| Difficulty stooping (scale 1-4^3^) | 2.11 | 2.09 | 0.39 |
| Difficulty walking (1/4 mile, scale 1-4^3^) | 44% | 43% | 0.34 |
| Difficulty dressing (scale 1-4^3^) | 17% | 16% | 0.13 |
| Difficulty eating scale 1-4^3^) | 9% | 9% | 0.24 |
| Self-rated health very good | 25% | 25% | 0.72 |
| Self-rated health good | 33% | 33% | 0.83 |
| Self-rated health fair | 20% | 20% | 0.98 |
| Self-rated health poor | 9% | 8% | 0.17 |
| Health self-rated as same as 1 year ago | 55% | 56% | 0.4 |
| Health self-rated worse than 1 year ago | 22% | 21% | 0.22 |
| Hearing aid | 12% | 12% | 0.34 |
| Institutional days^4^ | 32.69 | 31.19 | 0.48 |
| Inpatient hospital nights | 2.99 | 2.59 | 0.01 |
| Inpatient hospital stays | 46% | 40% | 0.00 |
| Height (cm) | 166.77 | 166.7 | 0.75 |
| Weight (kgs) | 71.57 | 71.68 | 0.76 |
| Probability death in the current calendar year^5^ | 8% | 5% | 0.00 |

**Note:** ^1^Some categories omitted as base/referent groups not shown: 8^th^ grade education or less, white, married, below poverty line, excellent health, health self-rated as better than 1 year ago. ^2^FPL=Federal Poverty Level. ^3^1= no/little difficulty, 2=some difficulty, 3= much difficulty, 4=unable to do. ^4^long-term care facility such as a nursing home, rehabilitation hospital, mental health facility, or institution for the developmentally disabled. ^5^Calculated by regressing actual deaths on covariates.

**Table S6: Cohort balance: Acute myocardial infarction**

| **Disease Categories**  **Table S7: Naïve and adjusted average cost and adjustment factors by disease** | **Naïve** | **Adjusted** | | **Coefficient(K)** | |
| --- | --- | --- | --- | --- | --- |
| **1-Infectious and parasitic diseases**  Immunizations and screening for infectious disease | $43 | $15 | | 0.35 | |
| Tuberculosis, STD, non-HIV, HIV, Other Infectious disease | $2,918 | $1,372 | | 0.47 | |
| **2-Neoplasms** |  |  | |  | |
| Colon cancer | $3,090 | $1,240 | | 0.40 | |
| Lung Cancer | $9,856 | $4,444 | | 0.45 | |
| Skin Cancer | $873 | $308 | | 0.35 | |
| Breast Cancer | $3,160 | $1,123 | | 0.36 | |
| Prostate Cancer | $2,591 | $988 | | 0.38 | |
| Hematologic Cancers | $8,196 | $3,431 | | 0.42 | |
| Benign Neoplasm | $134 | $47 | | 0.35 | |
| Cervical Cancer, Other Cancer | $3,023 | $1,143 | | 0.38 | |
| **3-Endocrine; nutritional; and metabolic diseases and immunity disorders** | | | | | |
| Thyroid Disorders | $1,152 | | $448 | 0.39 | |
| Diabetes Mellitus | $2,809 | | $1,119 | 0.40 | |
| Undiagnosed Diabetes Mellitus | $126 | | $43 | 0.34 | |
| Hyperlipidemia | $2,009 | | $720 | 0.36 | |
| Undiagnosed Hyperlipidemia | -$2,499 | | -$856 | 0.34 | |
| Gout and other crystal arthropathies | $975 | | $384 | 0.39 | |
| Other Endocrine Diseases | $3,264 | | $1,422 | 0.44 | |
| **4-Diseases of the blood and blood-forming organs** | | | | | |
| Anemias | $4,635 | $2,147 | | 0.46 | |
| Other Hematologic Disease | $4,824 | $2,328 | | 0.48 | |
| **5-Mental Illness** |  |  | |  | |
| ETOH Abuse, Illicit Drug Use, Tobacco Use | $2,782 | $1,188 | | 0.43 | |
| Dementia | $3,839 | $2,229 | | 0.58 | |
| Depression | $3,654 | $1,812 | | 0.50 | |
| Bipolar Disorder | $1,586 | $654 | | 0.41 | |
| Schizophrenia | $6,253 | $3,522 | | 0.56 | |
| Anxiety, Posttraumatic Stress Disorder (PTSD) | $1,054 | $450 | | 0.43 | |
| ADD-ADHD., Mental Retardation, Other Mental Health Disorders | $2,876 | $1,429 | | 0.50 | |
| **6-Diseases of the nervous system and sense organs** | | | | | |
| Otitis Media | $1,018 | $379 | | 0.37 | |
| Vestibular Disorders | $874 | $351 | | 0.40 | |
| Other Ear Disorders | $437 | $165 | | 0.38 | |
| Parkinson's Disease, Multiple Sclerosis, Paralysis | $3,524 | $1,635 | | 0.46 | |
| Seizure Disorders | $4,225 | $1,984 | | 0.47 | |
| Headaches, Migraine | $425 | $169 | | 0.40 | |
| Cataract | $413 | $146 | | 0.35 | |
| Glaucoma | $396 | $141 | | 0.36 | |
| Eye Disorders | $478 | $174 | | 0.37 | |
| Other Disease of the Central Nervous System (CNS) | $4,813 | $2,203 | | 0.46 | |
| **7-Diseases of the circulatory system** | |  | |  | |
| Hypertension | $3,541 | | $1,347 | 0.38 | |
| Undiagnosed Hypertension | -$2,917 | | -$897 | 0.31 | |
| Acute myocardial infarction (AMI) | $2,712 | | $1,142 | 0.42 | |
| Coronary Atherosclerosis and other heart disease | $3,790 | | $1,687 | 0.45 | |
| Atrial Fibrillation and Flutter, Other Arrhythmias | $2,584 | | $1,114 | 0.43 | |
| Cardiac Arrest (includes VF) | $4,986 | | $2,400 | 0.48 | |
|  | | | | | |
| **Disease Categories** | **Naïve** | | **Adjusted** | **Coefficient(K)** | |
| **7-Diseases of the circulatory system (Contd.)**  Congestive Heart Failure | $4,905 | | $2,514 | 0.51 | |
| Acute hemorrhagic stroke, Acute hemorrhagic stroke | $3,107 | | $1,593 | 0.51 | |
| Cerebrovascular Disease | $2,858 | | $1,341 | 0.47 | |
| Peripheral Vascular Disease | $2,289 | | $1,071 | 0.47 | |
| Other Cardiovascular Diseases | $3,106 | | $1,346 | 0.43 | |
| Other Vascular Diseases | $2,687 | | $1,137 | 0.42 | |
| Pulmonary embolism | $3,413 | | $1,593 | 0.47 | |
| DVT | $2,655 | | $1,200 | 0.45 | |
| **8-Diseases of the respiratory system** | |  | |  | |
| Pneumonia (non-TB, non-STD) | $4,116 | $2,318 | | 0.56 | |
| Influenza | $457 | $170 | | 0.37 | |
| Chronic Obstructive Pulmonary Disease | $3,212 | $1,477 | | 0.46 | |
| Asthma | $2,095 | $817 | | 0.39 | |
| Acute respiratory infection | $220 | $83 | | 0.38 | |
| Respiratory symptoms | $3,796 | $1,669 | | 0.44 | |
| Other Respiratory Diseases | $3,604 | $1,592 | | 0.44 | |
| **9-Diseases of the digestive system** | | | | | |
| Reflux/Ulcer Disease, Biliary Tract Disease, Liver Disease, Gastrointestinal Bleeding, Other Gastrointestinal Disorders | $3,438 | $1,370 | | 0.40 | |
| **10-Diseases of the genitourinary system** | | | |  | |
| Acute Renal Failure | $5,655 | $3,080 | | 0.54 | |
| Chronic Renal Failure, End Stage Renal Disease (ESRD) | $3,792 | $1,757 | | 0.46 | |
| UTI , Urinary Incontinence, Other Genitourinary Diseases | $2,572 | $1,020 | | 0.40 | |
| Hyperplasia of the Prostate | $510 | $187 | | 0.37 | |
| **11-Complications of pregnancy; childbirth; and the puerperium** | | | | | |
| Menopause, Pregnancy and Childbirth, Contraception | -$137 | -$45 | | 0.33 | |
| **12-Diseases of the skin and subcutaneous tissue** |  |  | |  | |
| Dermatologic Diseases | $1,279 | $509 | | 0.40 | |
| **13-Diseases of the musculoskeletal system and connective tissue** | | | |  | |
| Rheumatoid Arthritis | $2159 | $843 | | 0.39 | |
| Osteoarthritis | $727 | $277 | | 0.38 | |
| Back Pain | $928 | $349 | | 0.38 | |
| Osteoporosis | $786 | $307 | | 0.39 | |
| Other Rheumatic Diseases | $2589 | $1,024 | | 0.40 | |
| **14 -Congenital Anomalies & conditions originating in the perinatal period** | | | | | |
| Congenital Disorders, Newborn conditions | $3,080 | $1,272 | | 0.41 | |
| **15-Injury and poisoning** |  |  | |  | |
| Trauma, Fractures, Poisoning, Motor vehicle accident | $5,209 | $2,163 | | 0.42 | |
| Hip Fracture | $986 | $456 | | 0.46 | |
| **16-Other conditions** |  |  | |  | |
| Signs and Symptoms | $6,043 | $2,305 | | 0.38 | |
| Residual, unclassified, all other E codes | $2,790 | $1,146 | | 0.41 | |
| **17-Cancer Screening** |  |  | |  | |
| Screening: Breast Cancer | -$1,131 | -$350 | | | 0.31 |
| Screening: Colon Cancer | -$1,770 | -$561 | | | 0.32 |
| Screening: Prostate Cancer | -$2,830 | -$945 | | | 0.33 |
| Screening: Cervical Cancer | -$2,44 | -$691 | | | 0.29 |

**Table S7: Naïve and adjusted average cost and adjustment factors by disease (continued)**

1. SAS Institute Inc. SAS and all other SAS Institute Inc. product or service names are registered trademarks or trademarks of SAS Institute Inc., Cary, NC, USA. [↑](#footnote-ref-1)
